# Engineered, nucleocytoplasmic shuttling Cas13d enables highly efficient cytosolic RNA targeting

**DOI:** 10.1038/s41421-024-00672-1

**Published:** 2024-04-12

**Authors:** Christoph Gruber, Lea Krautner, Valter Bergant, Vincent Grass, Zhe Ma, Lara Rheinemann, Ariane Krus, Friederike Reinhardt, Lyupka Mazneykova, Marianne Rocha-Hasler, Dong-Jiunn Jeffery Truong, Gil Gregor Westmeyer, Andreas Pichlmair, Gregor Ebert, Florian Giesert, Wolfgang Wurst

**Affiliations:** 1 Institute of Developmental Genetics, Helmholtz Munich, Oberschleißheim, Germany; 2 TUM School of Life Sciences, Technical University of Munich, Freising, Germany; 3 Technical University of Munich, School of Medicine and Health, Institute of Virology, Munich, Germany; 4 Institute of Virology, Technical University of Munich/Helmholtz Munich, Munich, Germany; 5 Department of Bioscience, TUM School of Natural Sciences, Technical University of Munich, Munich, Germany; 6 Institute for Synthetic Biomedicine, Helmholtz Munich, Oberschleißheim, Germany; 7 TUM School of Medicine and Health, Technical University of Munich, Munich, Germany; 8 German Center for Infection Research (DZIF), Munich Partner Site, Munich, Germany; 9 Deutsches Zentrum für Psychische Gesundheit (DZPG), Site Munich-Augsburg, Germany; 10 German Center for Neurodegenerative Diseases (DZNE), Munich, Germany

**Keywords:** Protein transport, Molecular biology, Biological techniques

Dear Editor,

CRISPR/Cas13 systems are programmable tools for manipulating RNAs and are used in a variety of RNA-targeting applications^1–3^. Within the Cas13 family, Cas13d is the most active subtype in mammalian cells^4,5^. Recently, Cas13d was harnessed as an antiviral against diverse human RNA viruses^6,7^. However, Cas13d is barely active in the cytosol of mammalian cells, restricting its activity to the nucleus, which limits applications such as programmable antivirals^4,5^. Most RNA viruses replicate exclusively in the cytosol, suggesting that current Cas13d-based antivirals rely on uncontrolled nuclear leakage and are therefore limited in their efficiency^7^.

Here, we show that the nuclear localization of Cas13d crRNAs is the fundamental cause of Cas13d’s nuclear preference. To address this limitation, we engineered **n**ucleo**c**ytoplasmic **s**huttling Cas13d (Cas13d-NCS). Cas13d-NCS transfers nuclear crRNAs to the cytosol, where the protein/crRNA complex binds and degrades complementary target RNAs. We screened various designs of shuttling proteins and characterized multiple design parameters of the best-performing system. We show that Cas13d-NCS is superior for degrading mRNAs and a self-replicating RNA derived from the Venezuelan equine encephalitis (VEE) RNA virus. Ultimately, we harnessed Cas13d-NCS to completely block the replication of various SARS-CoV-2 strains. Cas13d-NCS, therefore, enables the rational manipulation of the subcellular localization of a CRISPR system.

**Fig. 1:**
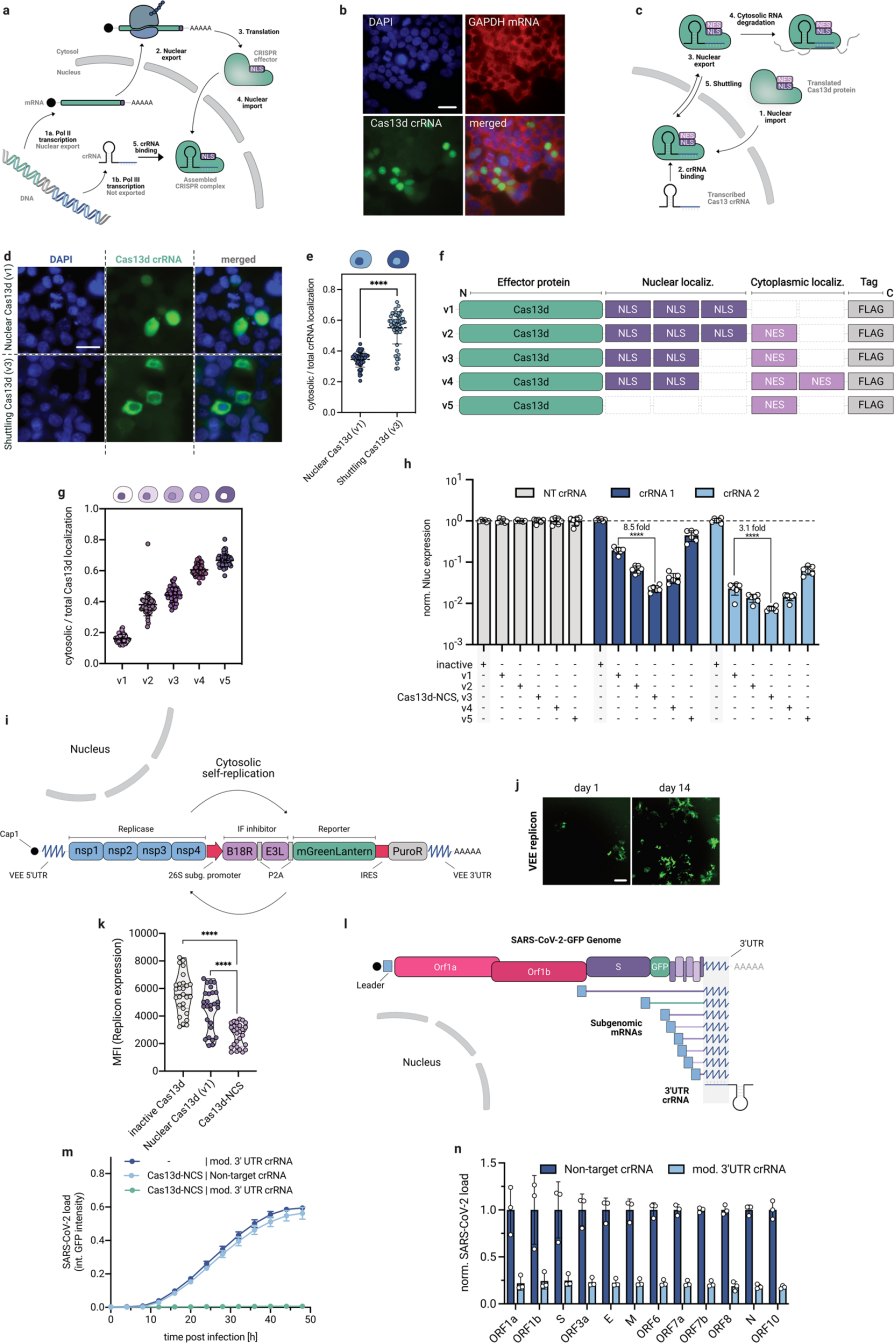
Engineering of Cas13d-NCS for efficient cytosolic RNA and antiviral targeting. **a** Schematic illustration of the expression and localization of Cas13d protein and crRNA in mammalians. Cas13d-NES has one C-terminal NES motif, corresponding to v5. **b** RNA FISH staining of the crRNA along with staining for GAPDH mRNA in cells expressing nuclear Cas13d-NLS (3xNLS, corresponds to v1 in **f**). Scale bar: 15 μm. **c** Schematic illustration of nucleocytoplasmic shuttling Cas13d to transport crRNAs to the cytosol. **d** RNA FISH staining of pol III expressed crRNAs, co-expressed with either nuclear (v1) or shuttling Cas13d (v3). Scale bar: 10 μm. **e** Quantification of cytosolic/total crRNA staining intensity for co-expressed nuclear and shuttling Cas13d. Unpaired Student’s *t*-test, ****P < 0.0001, mean ± s.d. for *n* = 50 cells. **f** Illustration of Cas13d shuttling constructs with varying NLS/NES motifs. **g** Quantification of cytosolic/total Cas13d protein staining intensity for different variants v1–v5 (*n* = 50 cells). **h** Comparison of nanoluciferase knockdown for Cas13d variants v1–v5. Unpaired Student’s *t*-test, ****P < 0.0001, mean ± s.d. for *n* = 6 biological replicates. **i** Schematic illustration of VEE reporter replicon. **j** Fluorescence imaging of replicon-expressing cells after short- and long-term cultivation. Scale bar: 30 μm. **k** Comparison of replicon targeting efficiency between nuclear and shuttling Cas13d-NCS using flow cytometry. Unpaired Student’s *t*-test, **P < 0.01; ****P < 0.0001, mean ± s.d. for 9 crRNAs, each measured in *n* = 3 biological replicates. **l** Schematic illustration of SARS-CoV-2-GFP reporter virus and viral transcriptome, targeted by a single 3’UTR crRNA. **m** Live measurement of SARS-CoV-2-GFP viral load by integrated GFP intensity under different targeting conditions (mean ± s.d., *n* = 4 biological replicates). **n** RNA-seq analysis of SARS-CoV-2 (Delta) subgenomic mRNA expression after 48 h of treatment with Cas13d-NCS and a non-target or 3’UTR target crRNA (*n* = 3 biological replicates).

To target RNA in mammalian cells by conventional Cas13d-NLS, Cas13d mRNA is transcribed by RNA polymerase II (pol II) and translated into the cytosol. Subsequently, the Cas13d protein is imported into the nucleus based on the nuclear localization sequence (NLS), where it forms a complex with the RNA polymerase III-transcribed crRNA. The protein/crRNA complex then degrades complementary target RNAs in the nucleus (Fig. 1a)^5^. Initially, we confirmed previous reports that Cas13d fused to NLS (Cas13d-NLS includes three C-terminal NLS motifs; corresponds to v1 in Fig. 1f) is more potent in knocking down target RNAs compared to Cas13d fused to a nuclear export sequence (NES; Cas13d-NES has one C-terminal NES motif; corresponds to v5 in Fig. 1f)^4,5^, and verified that both protein variants were correctly localized (Supplementary Fig. S1). Since mRNAs are rapidly exported to the cytosol after transcription, conventional Cas13d-NLS has a very short time window to recognize and bind its target mRNA in the nucleus. We thus speculated that Cas13d’s preference for nuclear localization and activity may be caused by the nucleus-restricted availability of crRNA, thus excluding the assembled complex from the cytosol. Staining of the crRNA revealed that it was indeed only present in the nucleus (Fig. 1b). This finding suggested that engineering a system for transporting crRNAs to the cytosol could shift Cas13d’s localization preference, which would enable applications requiring RNA targeting with subcellular precision.

For this, we explored various crRNA nuclear export strategies. Nuclear RNA export motifs^8^ or polymerase II promoter expression failed either due to insufficient crRNA export or reduced knockdown efficiency (Supplementary Fig. S2). Many natural systems transport cargos across the nuclear membrane by nucleocytoplasmic shuttling proteins^9,10^. Therefore, we explored the possibility that a nucleocytoplasmic shuttling Cas13d protein could transport crRNAs out of the nucleus. Such a shuttling Cas13d, fused to a sequence of both NLS and NES motifs, can be imported into the nucleus, bind to the crRNA, and be exported again to the cytosol in complex with the bound crRNA (Fig. 1c). Along with a crRNA, we expressed Cas13d, fused at the C-terminus to two NLS and one NES motif (v3 in Fig. 1f) and found that the system was indeed able to transport the crRNA to the cytosol (Fig. 1d, e). Next, we generated Cas13d variants with varying numerical ratios of the NLS and NES elements, as shown in the schematic in Fig. 1f. Characterization of the subcellular protein localization of these variants revealed that shuttling variants are semi-localized between nucleus and cytosol (Fig. 1g and Supplementary Fig. S3). We tested the efficiency of these variants to cleave transcripts of a co-transfected luciferase and found that all shuttling Cas13d variants improved the knockdown efficiency compared to conventional nuclear Cas13d. The optimal configuration v3, composed of two NLS and one NES, reduced the reporter expression by up to 99.3% and 8.5-fold over Cas13d-NLS (Fig. 1h). This variant (v3) is subsequently named Cas13d-NCS. We assume that Cas13d-NCS optimally balances sufficient nuclear import to bind crRNAs and sufficient nuclear export to efficiently target mainly cytosolic-localized mRNA. Furthermore, we found that Cas13d protein and crRNA are significantly reduced when expressed in separate cell compartments, suggesting an influence on the stability of the components (Supplementary Fig. S4).

We hypothesized that Cas13d-NCS is also superior for degrading viral cytosolic RNAs compared to conventional nuclear Cas13d-NLS since most RNA viruses transcribe their mRNAs and genomic RNAs exclusively in the cytoplasm^6^. To test this, we added an mGreenLantern reporter to a cytosolic, self-replicating RNA derived from the VEE virus (Fig. 1i). We in vitro transcribed the replicon RNA, transfected cells, selected and cultivated them. Subsequently, we confirmed stable replicon expression by fluorescence microscopy and solely RNA-based replication, without DNA intermediates, by RT-PCR (Fig. 1j and Supplementary Fig. S5a). We designed nine crRNAs, targeting different regions of the replicon RNA, and compared the knockdown efficiency of conventional Cas13d-NLS (v1) with shuttling Cas13d-NCS by flow cytometry (Supplementary Fig. S8). Remarkably, the knockdown was strong for Cas13d-NCS, but weak for Cas13d-NLS, confirming that Cas13d-NCS targets solely cytosolic RNA with greater efficiency compared to the current Cas13d system (Fig. 1k and Supplementary Fig. S5b).

Cas13d-NCS overcomes the limitation of sub-optimal localization of previously described Cas13d-based programmable antivirals^7^. To assess Cas13d-NCS antiviral efficacy, we targeted SARS-CoV-2 as a clinically relevant example. Since SARS-CoV-2 expresses subgenomic mRNAs through a discontinuous RNA synthesis mechanism, all viral mRNAs contain the genomic 3’untranslated region (UTR)^11^. We targeted this 3’UTR with a single crRNA to degrade all viral genomic and transcript RNA directly (Fig. 1l). To demonstrate the therapeutic potential of Cas13d-NCS antivirals, we developed and tested an RNA-based expression system consisting of an optimized Cas13d-NCS mRNA scaffold, a chemically stabilized crRNA and formulated both components in a lipid–RNA complex (Supplementary Fig. S6a, b). First, we confirmed that RNA expressed Cas13d-NCS is functional, by knocking down a SARS-CoV-2 reporter RNA by 96% (Supplementary Fig. S6c). To verify the targeting accuracy of Cas13d-NCS, we analyzed the expression level of the five most likely off-target genes. Levels in cells either treated with Cas13d-NCS and the SARS-CoV-2 3’UTR crRNA or a non-target control crRNA showed no significant differences (Supplementary Fig. S6d). Next, we delivered Cas13d-NCS mRNA along with different crRNAs to cells, infected them with SARS-CoV-2-GFP^12^, and measured the GFP expression as a measure of viral load over time. Interestingly, targeting conserved but weakly expressed viral-coding sequences resulted in relatively weak inhibition, whereas targeting the ubiquitous 3’UTR with a single crRNA resulted in complete inhibition of viral replication (Supplementary Fig. S7a, b). Furthermore, real-time fluorescent microscopy tracking the infection progression for 48 h showed that Cas13d-NCS entirely prevented the replication of SARS-CoV-2-GFP reporter virus (Fig. 1m). Furthermore, we confirmed that Cas13d-NCS/3’UTR crRNA efficiently inhibited the replication and expression of all viral transcripts of the severe SARS-CoV-2-Delta variant both when applied 24 h before (Fig. 1n and Supplementary Fig. S7c), as well as 3, 6, and 9 h after viral infection (Supplementary Fig. S7d). The flexibility and high efficiency of Cas13d-NCS demonstrate its great potential as a programmable therapy against cytosolic RNA viruses.

In our study, we demonstrated that conventional Cas13d applications are limited to the nucleus due to crRNA localization. In contrast, Cas13d-NCS transports crRNAs out of the nucleus by shuttling between the nucleus and cytosol. Previous attempts to redirect the Cas13d system to the cytosol by fusing an NES did not take crRNA localization into account or relied on uncontrolled nuclear leakage of the crRNA/protein complex^4,5,7^. Other CRISPR systems, dependent on nuclear-transcribed gRNAs, could benefit from the described crRNA transport framework as well by applying similar engineering principles to these systems. In summary, we envision that Cas13d-NCS will unleash the full potential of RNA targeting with subcellular precision and enable the development of novel molecular tools and therapies for RNA-related diseases.

### Supplementary information

Supplementary information, Figures and Tables
