# Supplementary material for: Engineered, nucleocytoplasmic shuttling Cas13d enables highly efficient cytosolic RNA targeting
Source: Cell Discov. 2024 Apr 12;10:42. doi: 10.1038/s41421-024-00672-1 (PMC11015019; doi:10.1038/s41421-024-00672-1)
Supplement: Supplementary file 1 — Supplementary information, Figures and Tables [file 41421_2024_672_MOESM1_ESM.pdf]

# Engineered, nucleocytoplasmic shuttling Cas13d enables highly efficient cytosolic RNA targeting

## Supplementary Information

### Materials and Methods

### Methods

**Molecular cloning**

Mammalian codon optimized RfxCas13d coding sequence was synthesized (IDT) and cloned into a CAG promoter and bGH containing mammalian expression plasmid. Additionally, a FLAG tag and different NLS/NES motifs were synthesized (IDT) and added to the C-terminus of Cas13d (Suppl. Tab. 2). BsbI flanked crRNA expression cassette was synthesized (IDT) and cloned into a U6 promoter containing plasmid. Subsequently, different spacer sequences were inserted by BbsI (NEB) digest of the backbone and ligation of annealed oligonucleotides (Metabion, Suppl. Tab. 1). All clonings were performed using standard molecular techniques, using Q5 DNA polymerase (NEB), restriction enzymes (NEB), NEBuilder HiFi DNA Assembly (NEB), Instant Sticky-end Ligase Master Mix (NEB) and self-made chemically competent *E. coli* DH5alpha cells.

**Cell culture**

HEK293T cells were cultivated at 37°C, 5 % $CO_2$ in an $H_2O$-saturated atmosphere, and maintained in DMEM (Gibco) supplemented with 10% FBS (Gibco) and 1% penicillin-streptomycin (Gibco). For generating HEK293T-ACE2 cells, the ACE2 sequence was amplified from an ACE2 expression vector (provided by S. Pöhlmann) and cloned into the lentiviral vector pWPI-puro. Subsequently, HEK293T cells were transduced and selected with puromycin.

**Plasmid transfection**

The day before transfection, cells were seeded at 2.5 x$10^4$ cells per well for 96 well, 7 x$10^4$ cells per well for 48 well, 1.5 x$10^5$ for 24 well, and 5.0 x$10^4$ cells per well for 8-well slides. Cells were transfected with JetOptimus DNA transfection reagent (Polyplus transfection). For 96 well and 8-well format, 75 ng DNA per well was transfected, and for 48 well, 150 ng DNA per well. Cells were analyzed or further processed after 2-3 days.

**Knockdown of TFRC**

Samples were transfected in a 48 well plate with different Cas13d targeting constructs. Three days after transfection, the medium was removed, and cells were detached using Accutase (Gibco). Samples were then transferred into tubes and centrifuged for 10 min. Subsequently, cells were resuspended in staining solution containing TFRC antibody BLD-334105 (Biozol), diluted 1:2,000 in 1xPBS with 5% (v/v) FBS. Cells were incubated for 30 min. at 4°C, washed 2x with PBS, transferred to FACS tubes, and analyzed by flow cytometry for TFRC expression (BD FACSaria II).

**Imaging and quantification of Cas13d variants localization**

48 h post-transfection, Cas13d transfected cells were fixed with 10 % formalin-solution (Sigma-Aldrich), washed with PBS, permeabilized (1% BSA, 0.5% Triton X-100 in PBS), and stained with 1:500 diluted M2 FLAG primary antibody (F1804, Merck) and 1:1,000 diluted secondary antibody (A21203,

Thermo Fisher). Additionally, cellular membranes were stained with Wheat Germ Agglutinin (CF488A, Thermo Fisher). Subsequently, cells were stained with DAPI and imaged using EVOS Cell Imaging (Thermo Fisher) or CellInsight NTX High Content Analysis (Thermo Fisher). Nuclear and cytosolic fluorescence intensities were analyzed in ImageJ using the measurement tool.

**Fluorescence *in situ* hybridization (FISH) analysis of crRNA and GAPDH mRNA localization**

In an 8-well slide transfected cells were fixed with 10 % formalin solution (Sigma-Aldrich) and washed 3x with PBS. Subsequently, cells were permeabilized with ice-cold 70 % ethanol at 4°C for 4 h, incubated with pre-hybridization-solution (2x saline sodium citrate (SSC) buffer, 10% formamide, 1:20 ribonucleoside vanadyl complex (VRC, NEB) for 15 min. at 37°C. 5' and 3' Atto488 labeled crRNA probe (Metabion) and Quasar labeled GAPDH probe (Stellaris) were diluted to a final concentration of 10 ng/µl in FISH hybridization buffer (dd$H_2O$, 2x SSC, 10 % formamide, 15 mg/ml tRNA, 50 % dextran sulfate, 50 mg/ml BSA, 10 mM VRC) and incubated at 37°C overnight. The next day, samples were washed 3x with pre-hybridization buffer, followed by 3x washing with PBS and DAPI staining. Cells were imaged using an Axioplan 2 microscope (Zeiss) or EVOS Cell Imaging (Thermo Fisher) device, and fluorescence intensities in cytosol and nucleus were analyzed in ImageJ using the measurement tool.

**Knockdown of nanoluciferase reporter**

Cells were transfected in a 96 well plate with Cas13d constructs and nanoluciferase, fused to mouse ornithine decarboxylase PEST, coding plasmid. 48 h post transfection cells were lysed using 5X passive Lysis buffer (Promega). 10 µl lysate was diluted in 40 µl PBS, mixed with NanoGlo assay (Promega) and luminescence was measured at a Centro LB 960 (Berthold) plate reader.

**Western Blot analysis of protein stability**

Transfected cells were lysed with M-PER Mammalian Protein Extraction Reagent (Thermo Fisher), denatured with Laemmli buffer at 95°C (Sigma Aldrich), and run on a 15-4 % gradient SDS gel (Invitrogen) in NuPAGE Running Buffer (Thermo Fisher). Proteins were blotted onto a polyvinylidene fluoride (PVDF) membrane (Bio-Rad) overnight at 4°C and 15 V. The membrane was blocked and incubated at 4°C overnight in 5 % milk powder solution with 1:1,000 diluted primary M2 FLAG antibody (F1804, Merck). Subsequently, the membrane was washed, incubated with peroxidase-conjugated mouse secondary antibody (115-035-003, Dianova) together with peroxidase-coupled GAPDH antibody (GTX627408, GeneTex), washed again and imaged with Amersham ECL Prime Detection Reagent (Sigma-Aldrich) using a Fusion FX device (Vilber).

**VEE replicon cell line generation**

In T7-VEE-OKS-iM plasmid (Addgene #58972, a gift from Dr. Steven Dowdy), inserts were replaced (except for the puromycin resistance gene) by B18R and E3L genes (Twist Bioscience) derived from VEE virus to extend the persistence of the replicon. Additionally, a mGreenLantern reporter gene (IDT, Suppl. Tab. 3) was added. The plasmid was linearized by MluI digest and subsequently *in vitro* transcribed with HiScribe T7 Quick High Yield RNA Synthesis Kit (NEB). The RNA was 5' capped with Vaccina Capping System along with 2´-O-Methyltransferase (NEB) and 3' polyadenylated using *E. coli* Poly(A) Polymerase (NEB). The mRNA was transfected using JetMessenger RNA transfection reagent (Polyplus transfection), and after 72 h, cells were selected with 1 µg/ml puromycin (Gibco). On days 1 and 14, RNA replication was confirmed by imaging the mGreenLantern reporter protein. Additionally, RNA-dependent replication without DNA intermediate was confirmed by comparing PCR to RT-PCR (SuperScript IV One-Step, Thermo Fisher) amplification of extracted RNA (Monarch Total RNA Isolation

Kit, NEB) or DNA (Wizard Genomic DNA Purification Kit, Promega) for VEE and a control primer set for the actively transcribed AAVS1 genomic locus.

**Knockdown measurement of VEE replicon**

VEE replicon expressing cells were transfected with different Cas13d and crRNA constructs. After 72 h, cells were detached, and mGreenLantern, was analyzed by flow cytometry.

**Optimization of RNA-based Cas13d-NCS system**

Cas13d-NCS-P2A-mRuby3 coding sequence (IDT, Suppl. Tab. 3) was cloned into plasmid backbones with or without alpha-Globin 5'UTR (Twist Bioscience), 2x beta-Globin (amplified from gDNA), 60xA polyA sequence (available in the lab), and a T7 promoter. The cloned plasmids were linearized by restriction digest and *in vitro* transcribed with or without N1-methyl-pseudouridine (Jena Bioscience). 5' and 3' 2'O'Methyl Phosphotiodate modified crRNA was ordered from IDT. Templates for *in vitro* transcribed crRNAs were generated by annealing and elongating complementary oligos containing T7 promoter and the crRNA sequence. Elongation products were used directly for *in vitro* transcription with HiScribe T7 Quick High Yield RNA Synthesis Kit (NEB) and subsequently purified with Monarch RNA Cleanup kit (NEB). 100 ng Cas13d-NCS-P2A-mRuby3 and 50 ng crRNA were transfected using JetMessenger RNA transfection reagent (Polyplus transfection). After 24 h, cells were imaged using an EVOS Cell Imaging device (Thermo Fisher). For knockdown experiments, UTR containing, modified Cas13d-NCS coding mRNA was transfected together either with either unmodified or chemically stabilized crRNA and a nanoluciferase reporter RNA, containing a fragment of the SARS-CoV-2 3'UTR. After 24 h, reporter knockdown was measured (NanoGlo assay, Promega) at a Centro LB 960 (Berthold) plate reader.

**Off-target analysis**

Potential off-target transcripts were identified by performing a BLAST search for the sequence of the 3'UTR crRNA (s. Supplementary Table 1) in the human transcriptome. For the top 5 hits, primer sets were designed and ordered (IDT). HEK293T Cells were transfected with Cas13d-NCS mRNA and crRNAs as described before. 24 h after the transfection, RNA was isolated (Monarch RNA Miniprep Kit, NEB) and a RT-qPCR was performed (Luna Universal One-Step RT-qPCR Kit, NEB).

**Viral strains, stock preparation and plaque assay**

SARS-CoV-2 (delta) and SARS-CoV-2-GFP strains were produced by infecting Vero E6 cells cultured in DMEM medium (10% FCS, 100 µg/ml streptomycin, 100 µg/ml penicillin) for 2 days (MOI of 0.01). Viral stocks were collected and spun twice (1,000 g for 10 min.) before storage at −80 °C. The titer of viral stock was determined by plaque assay. Confluent monolayers of Vero E6 cells were infected with serial five-fold dilutions of virus supernatants for 1 h at 37 °C. The inoculum was removed and replaced with serum-free DMEM (Gibco) containing 0.5% carboxymethylcellulose (Sigma-Aldrich). Two days after infection, cells were fixed for 20 min. at room temperature with formaldehyde added directly to the medium to a final concentration of 5%. Fixed cells were washed extensively with PBS before staining with water containing 1% crystal violet and 10% ethanol for 20 min. After rinsing with PBS, the number of plaques was counted, and the virus titer was calculated.

**SARS-CoV-2-GFP reporter virus assay**

HEK293T-ACE2 cells were seeded into 96 well plates 1 day before transfection. Subsequently, cells were transfected with Cas13d-NCS mRNA (100 ng/well) and crRNAs (50 ng/well) using JetMessenger

reagent (Polyplus transfection), one day before or 3-9h after infection respectively. RNAs were synthesized as described in the previous section. Immediately following infection with SARS-CoV-2-GFP (MOI 3, applied from a master mix in equal amounts to all treatment and control conditions), the plate was placed in the IncuCyte S3 Live-Cell Analysis System (Sartorius), where images of phase, green, and red channels were captured at regular time intervals at 4× magnification. Cell viability was assessed as cell confluence per well (phase area). Virus growth was assessed as GFP integrated intensity normalized to cell confluence per well (GFP integrated intensity/phase area). Basic image analysis and image export were performed using the IncuCyte S3 software (Essen Bioscience; version 2019B Rev2).

**Comparison of fluorescence and RT-qPCR based quantification of viral load**

Vero E6 cells were transfected with Cas13d-NCS coding mRNA and crRNA as described before. 24 h after the transfection, cells were infected with SARS-CoV-2-GFP (MOI 1), and GFP expression was monitored for 24 h in the IncuCyte S3. Subsequently, RNA was isolated by QIAZol extraction (Qiagen) and the viral load was analyzed by RT-qPCR (2019-nCoV RUO Kit, IDT and Luna Universal Probe One-Step RT-qPCR, NEB).

**Analysis of therapeutic treatment of infected cells**

Vero E6 cells were seeded in 96 well format and 24 h later infected with SARS-CoV-2-GFP (MOI 1). Infected cells were then transfected with Cas13d-NCS coding mRNA and crRNA at different time points after the infection. Viral replication was monitored by GFP intensity in the IncuCyte S3 for 48 h.

**SARS-CoV-2 delta infection and expression analysis**

HEK293T-ACE2 cells were seeded into 24 well plates. One day post-seeding, the cells were transfected with 400 ng of Cas13d-NCS mRNA and 200 ng of crRNAs per well using JetMessenger reagent (Polyplus transfection). RNAs were synthesized as described in the previous section. One day post-transfection, the medium was exchanged prior to infection with SARS-CoV-2 delta strain at MOI 0.5. RNA was isolated from infected cells by QIAZol extraction (Qiagen) 48 h post-infection. RNA sequencing library preparation and Illumina paired-end sequencing were performed by GENEWIZ (Leipzig, Germany). Sequencing reads were processed in Geneious Prime by mapping them to the SARS-CoV-2 reference sequence NC_045512 and performing differential expression analysis.

**Statistical Analysis**

Statistical tests and graphical representation of numerical data were performed in GraphPad Prism 8.

## Supplementary Figures

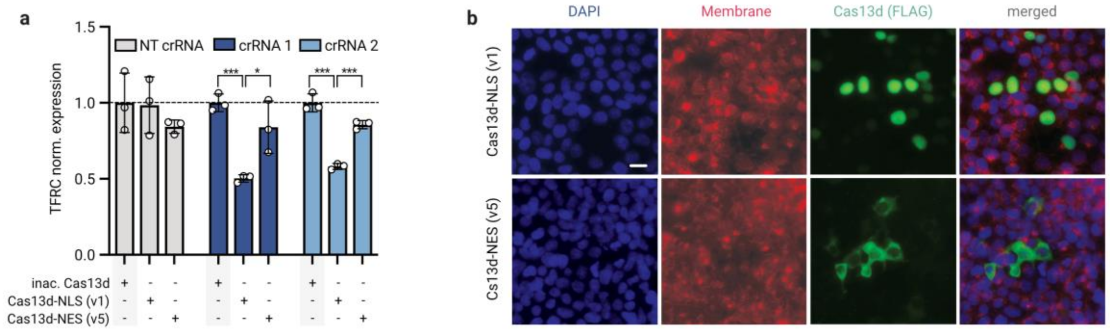


**Supplementary Fig. S1 | Characterization of Cas13d-NLS (v1) and -NES (v5). a,** Flow cytometry analysis of TFRC knockdown for nuclear and cytosolic Cas13d. (Unpaired Student's t-test with *P<0.05; ***P<0.001, mean +/- s.d. for n=3 biological replicates). **b,** Immunofluorescence staining and imaging of NLS- or NES-fused Cas13d subcellular localization. Scale bar: 15 µm.

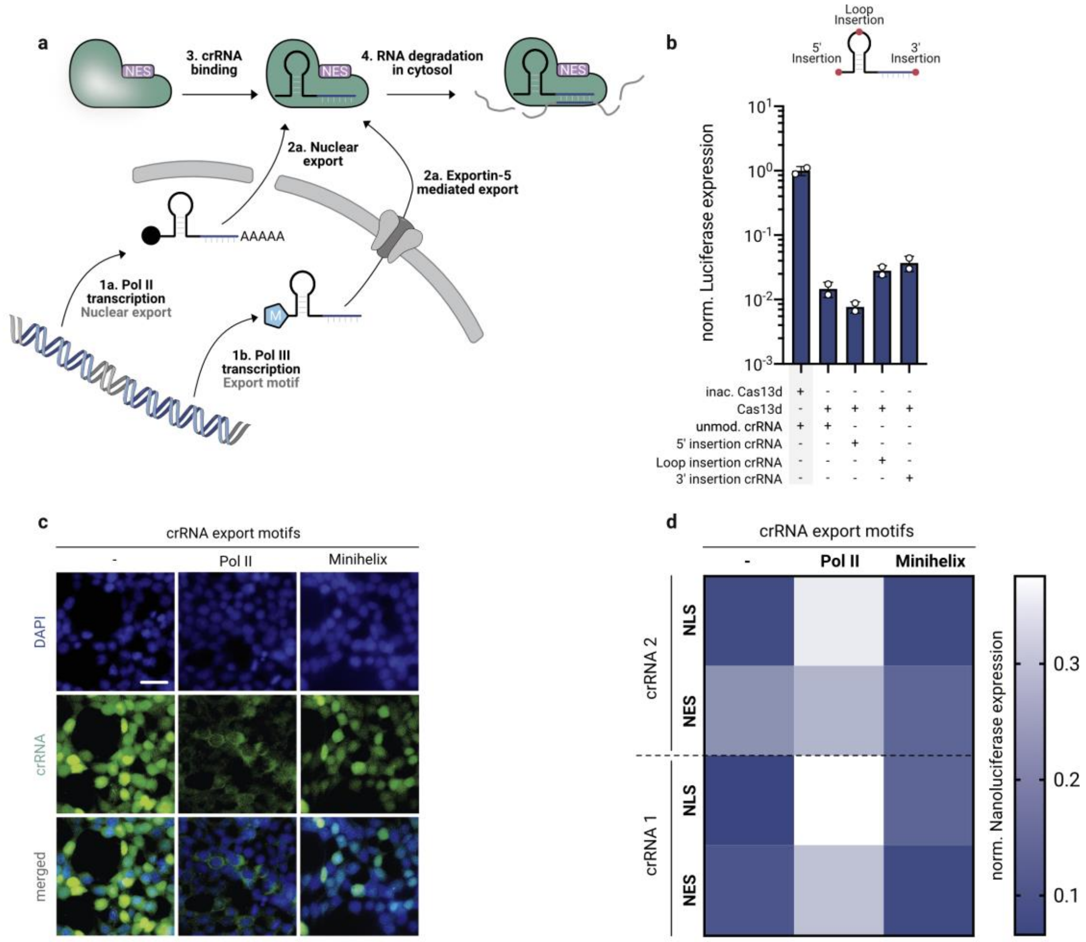


**Supplementary Fig. S2 | Development of nuclear crRNA export strategies. a,** Schematic illustration of different crRNA export strategies based on viral export motifs or endogenous mRNA export machinery. **b,** Knockdown efficiency against nanoluciferase reporter for crRNAs containing 5', 3', or loop insertions of a random RNA motif (n=2 biological replicates). **c,** RNA FISH staining to test for subcellular localization of pol II expressed and minihelix modified crRNA. Scale bar: 20 µm. **d,** Knockdown efficiency against nanoluciferase of pol II expressed or minihelix modified crRNAs in combination with either nuclear or cytosolic localized Cas13d protein (n=6 biological replicates).

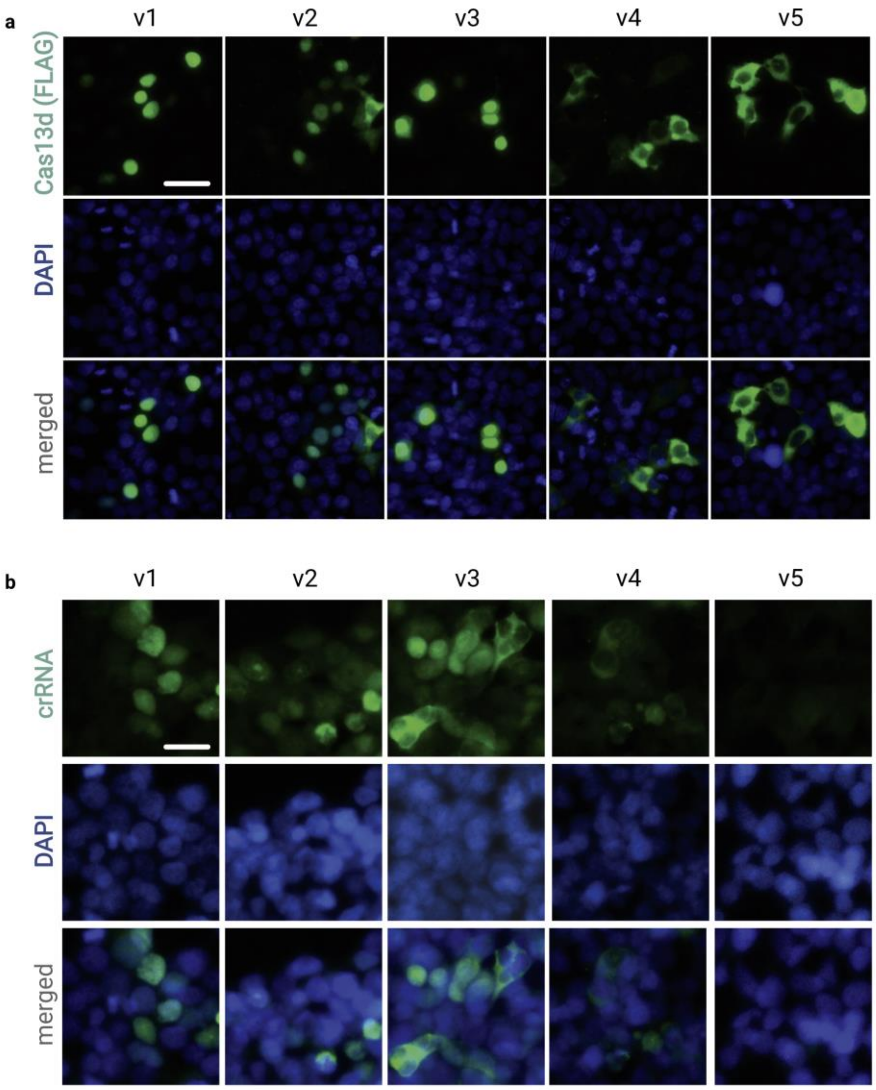


**Supplementary Fig. S3 | Analysis of protein and crRNA localization for Cas13d variants v1-v5. a,** Characterization of Cas13d protein localization for different combinations of NLS and NES signals by antibody staining for Cas13d. Scale bar: 20 µm. **b,** Characterization of crRNA localization when co-expressed with differentially localized Cas13d variants. Scale bar: 15 µm

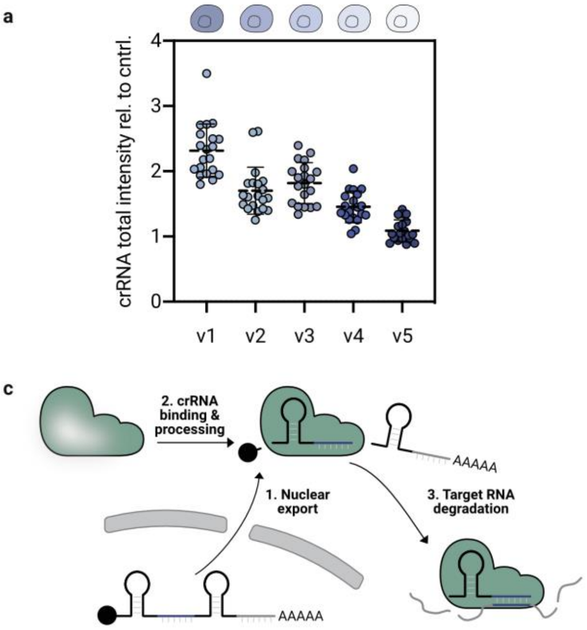


**Supplementary Fig. S4 | Co-dependency of Cas13d crRNA and protein stability. a,** Analysis of crRNA stability by RNA FISH staining and fluorescence intensity measurement for co-expressed shuttling Cas13d variants (n=20 cells, normalized to unstained cells). **b,** Western Blot of Cas13d v1-v5, co-transfected with a pol III expressed crRNA. **c,** Schematic illustration of pol II crRNA export and processing. **d,** Western Blot of Cas13d v1-v5, co-transfected with a pol II expressed crRNA.

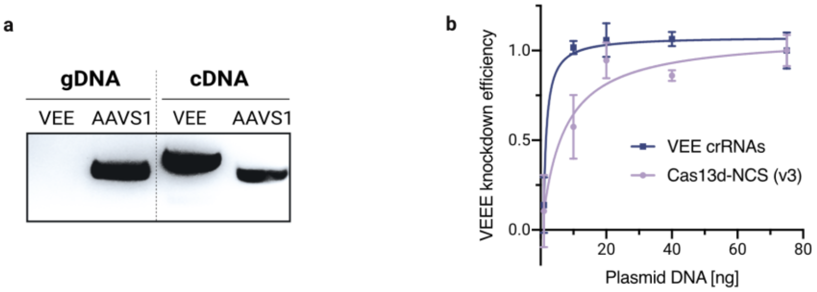


**Supplementary Fig. S5 | VEE replicon and targeting characterization. a,** Confirmation of solely RNA-based replicon replication by RT-PCR and PCR amplification of isolated RNA and genomic DNA. **b,** Optimization of crRNA and Cas13d-NCS coding plasmid concentrations for VEE replicon targeting, n=3 biological replicates.

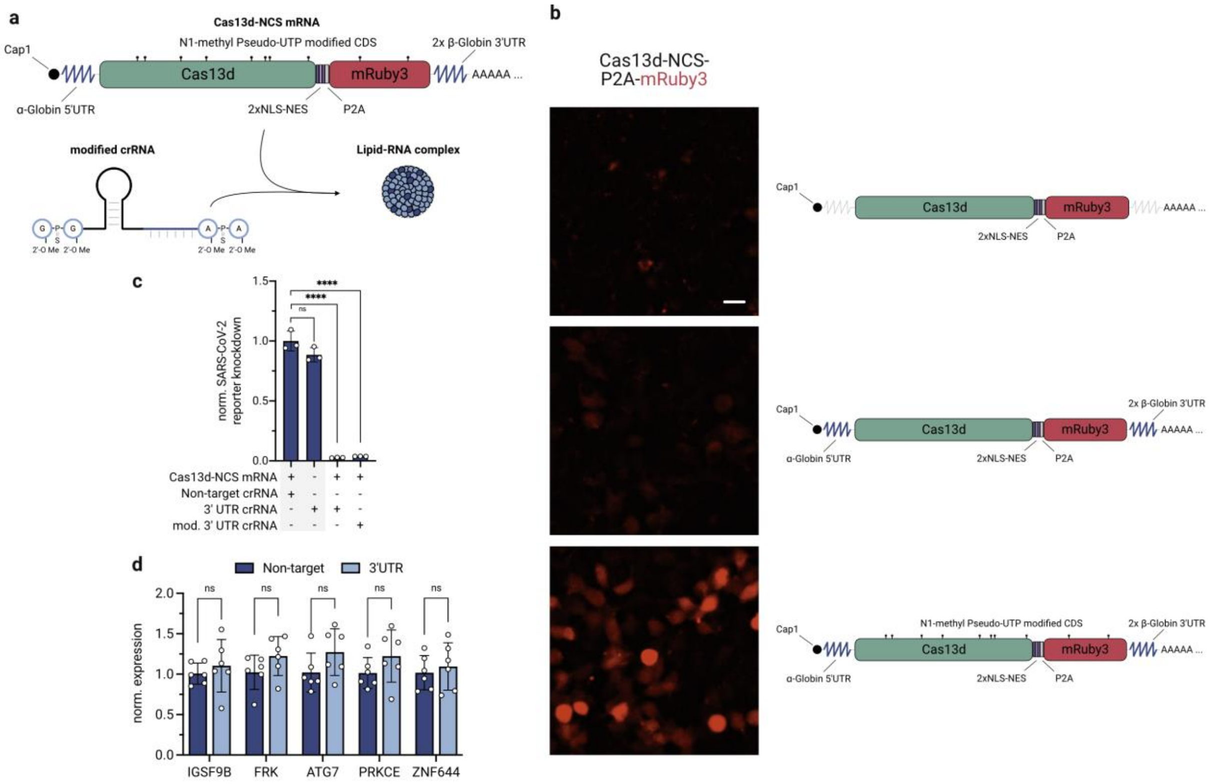


**Supplementary Fig. S6 | Establishing an RNA-based Cas13d-NCS system. a,** Schematic representation of optimized Cas13d-NCS mRNA and chemically stabilized crRNA, formulated in a lipid-RNA complex. **b,** Expression of Cas13d-NCS-P2A-mRuby3, imaged by fluorescence microscopy, for an unmodified, UTR stabilized and N1-methyl Pseudo-UTP modified mRNA. Scale bar: 15 µm. **c,** Knockdown measurement of RNA-encoded Cas13d-NCS against a nanoluciferase reporter RNA with cloned SARS-CoV-2 3'UTR. (Unpaired Student's t-test, ****P<0.0001, mean +/- s.d. for n=3 biological replicates). **d,** Analysis of potential off-target effect of Cas13d-NCS by RT-qPCR for top 5 predicted off-target transcripts. (Unpaired Student's t-test for n=6 biological replicates).

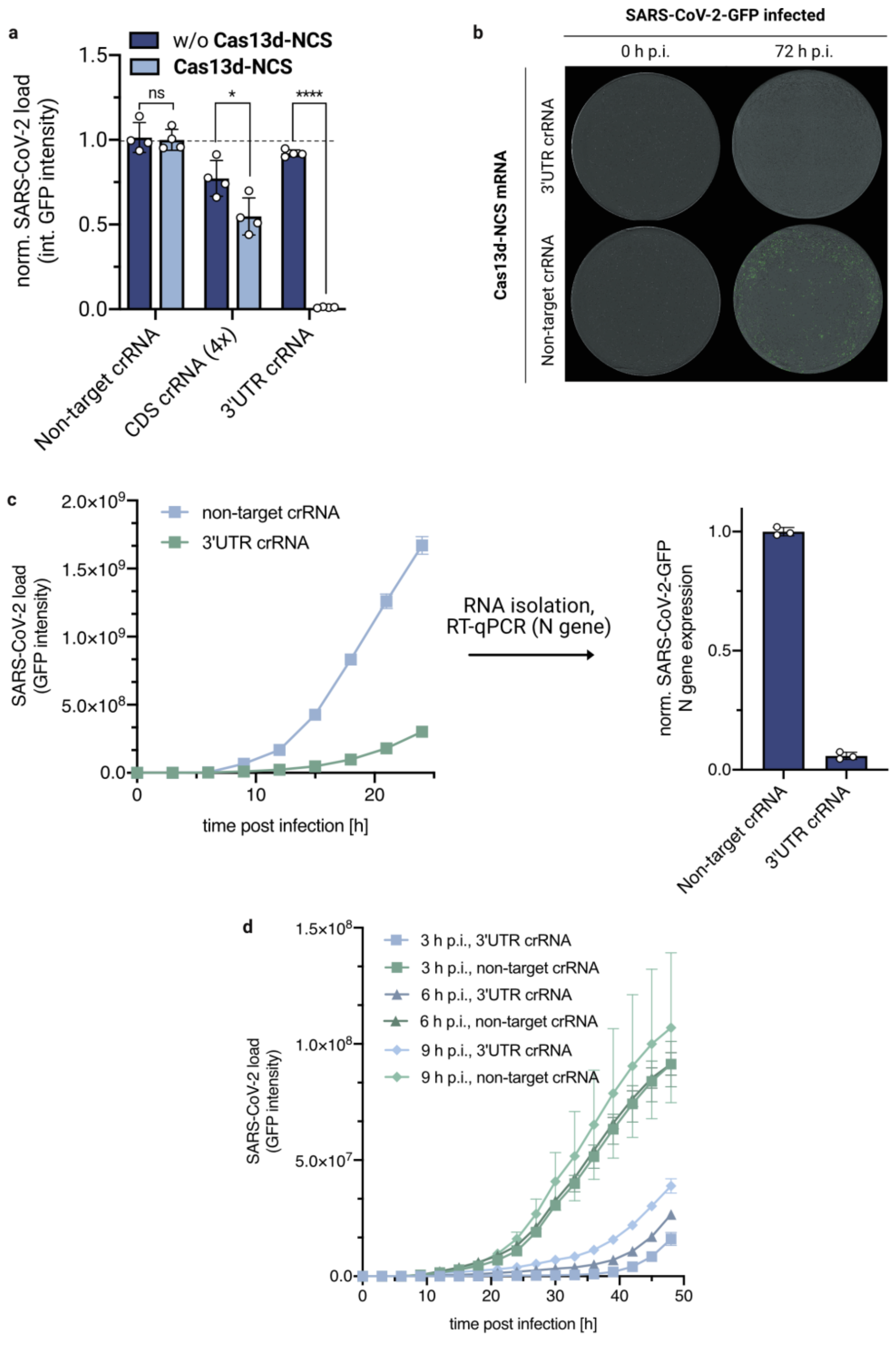


**Supplementary Fig. S7 | Targeting of SARS-CoV-2 with Cas13d-NCS. a,** SARS-CoV-2-GFP replication, measured by integrated GFP intensity after 48 h for either a pool of 4 crRNAs targeting coding sequences, or a single crRNA targeting the 3'UTR. (Unpaired Student's t-test, *P<0.05; ****P<0.0001, mean +/- s.d. for n=4 biological replicates). **b,** Exemplary images of SARS-CoV-2 infected cells at two different time points (p.i., post infection) during treatment with Cas13d-NCS. **c,** Methodological comparison of Cas13d-NCS induced antiviral effect, measured by GFP intensity and RT-qPCR for n=3 biological replicates. **d,** Therapeutic treatment of infected cells by transfecting Cas13d-NCS 3-9 h post infection (p.i.) for n=3 biological replicates.

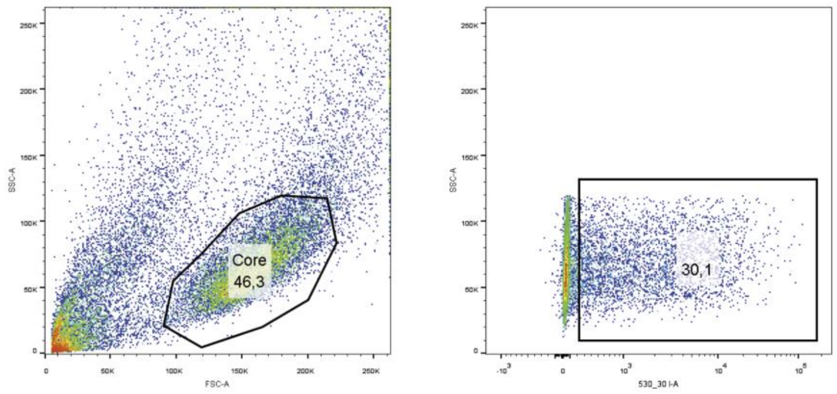


**Supplementary Fig. S8 |** Exemplary FACS gating scheme for live/single cells (left panel) and mGreenLantern replicon expressing cells (right panel)

**Supplementary Table 1 |** crRNAs used in this study

| | |
|---|---|
| Nluc 1-2 | GATCTTCAGGCCGTTCTCGCCGCTCAGCAC<br>CTGGATAGGGGTCACAGACACTCCCAGGTT |
| mRuby3 | ATGGTCTGCACGCCCTCATAAGGTCTGCCC |
| mGL replicon 1-9 | GTAACCTTTATCAAGCCACGAGGTGT<br>GTAACCTTTATCAAGCCACGAGGTGT<br>ACCTAGCAAAACATGCGACACCATAC<br>TGGGCTTCTCTCATGCGCCGCCCATC<br>AGGTAATTGGTCTGGGCTTCTCTCAT<br>AAAATAAAAATTTTAAGGCGGCATGC<br>GCGAGTTCTATGTAAGCAGCTTGCCA<br>CAGGCAACAAAACTGGTCCATCCCCA<br>AGGCAACAAAACTGGTCCATCCCCAA |
| SARS-CoV-2 CDS 1-4 | CCACATAATAAGCTGCAGCACCAGCTGT<br>GCATTAATACAGCCACCATCGTAACAAT<br>ACAGAGATTATAAGAGCCCACATGGAAA<br>GGTGCATGTAACAAAAAGAGACACAGTC |
| SARS-CoV-2 3'UTR (mod.) | TTAAAATCACATGGGGATAGCACTACTA |
| TFRC 1-2 | CCTCACAAATGAAAGCAGTTGGCTGTTG<br>TCCACGAGCAGAATACAGCCACTGTAAA |
| Nluc 1-2, (+5'/3'/loop insertion in DR) | GATCTTCAGGCCGTTCTCGCCGCTCAGCAC<br>CTGGATAGGGGTCACAGACACTCCCAGGTT |
| mRuby3 (in pol II BB) | ATGGTCTGCACGCCCTCATAAGGTCTGCCC |

**Supplementary Table 2 |** Cas13d proteins used in this study

Cas13d, NLS sequences, NES sequences, linker and tag

| | |
|---|---|
| V1 (Nuclear Cas13) | MIEKKKSFAKGMGVKSTLVSGSKVYMTTFAEGSDARLEKIVEGDSIRSVNEGEAFSA<br>EMADKNAGYKIGNAKFSHPKGYAVVANNPLYTGPVQQDMLGLKETLEKRYFGESA<br>DGNDNICIQVIHNILDIEKILAEYITNAAYAVNNISGLDKDIIGFGKFSTVYTYDEFKDPE<br>HHRAAFNNNDKLINAIKAQYDEFDNFLDNPRLGYFGQAFFSKEGRNYIINYGNECYD<br>ILALLSGLRHWVVHNNEEESRISRTWLYNLDKNLDNEYISTLNYLYDRITNELTNSFSK<br>NSAANVNYIAETLGINPAEFAEQYFRFSIMKEQKNLGFNITKLREVMLDRKDMSEIR<br>KNHKVFDSIRTKVYTMMDFVIYRYYIEEDAKVAAANKSLPDNEKSLSEKDIFVINLRG<br>SFNDDQKDALYYDEANRIWRKLENIMHNIKEFRGNKTREYKKKDAPRLPRILPAGRD<br>VSAFSKLMYALTMFLDGKEINDLLTTLINKFDNIQSFLKVMPLIGVNAKFVEEYAFFK<br>DSAKIADELRLIKSFARMGEPIADARRAMYIDAIRILGTNLSYDELKALADTFSLDENG<br>NKLKKGKHGMRNFIINNVISNKRFHYLIRYGDPAHLHEIAKNEAVVKFVLGRIADIQK<br>KQGQNGKNQIDRYYETCIGKDKGKSVSEKVDALTKIITGMNYDQFDKKRSVIEDTGR<br>ENAEREKFKKIISLYLTVIYHILKNIVNINARYVIGFHCVERDAQLYKEKGYDINLKKLEE |

| | |
|---|---|
| | KGFSSVTKLCAGIDETAPDKRKDVEKEMAERAKESIDSLESANPKLYANYIKYSDEKKA EEFTRQINREKAKTALNAYLRNTKWNVIIREDLLRIDNKTCTLFRNKAVHLEVARYVH AYINDIAEVNSYFQLYHYIMQRIIMNERYEKSSGKVSEYFDAVNDEKKYNDRLLKLLC VPFGYCIPRFKNLSIEALFDRNEAAKFDKEKKKVSGNSGSTPPKKKRKVEDGEGPAAK RVKLDSGAAPAAKKKKLDYKDDDDK |
| V2 | MIEKKKSFAKGMGVKSTLVSGSKVYMTTFAEGSDARLEKIVEGDSIRSVNEGEAFSA EMADKNAGYKIGNAKFSHPKGYAVVANNPLYTGPVQQDMLGLKETLEKRYFGESA DGNDNICIQVIHNILDIEKILAEYITNAAYAVNNISGLDKDIIGFGKFSTVYTYDEFKDPE HHRAAFNNNDKLINAIKAQYDEFDNFLDNPRLGYFGQAFFSKEGRNYIINYGNECYD ILALLSGLRHWVVHNNEEESRISRTWLYNLDKNLDNEYISTLNYLYDRITNELTNSFSK NSAANVNYIAETLGINPAEFAEQYFRFSIMKEQKNLGFNITKLREVMLDRKDMSEIR KNHKVFDSIRTKVYTMMDFVIYRYYIEEDAKVAAANKSLPDNEKSLSEKDIFVINLRG SFNDDQKDALYYDEANRIWRKLENIMHNIKEFRGNKTREYKKKDAPRLPRILPAGRD VSAFSKLMYALTMFLDGKEINDLLTTLINKFDNIQSFLKVMPLIGVNAKFVEEYAFFK DSAKIADELRLIKSFARMGEPIADARRAMYIDAIRILGTNLSYDELKALADTFSLDENG NKLKKGKHGMRNFIINNVISNKRFHYLIRYGDPAHLHEIAKNEAVVKFVLGRIADIQK KQGQNGKNQIDRYYETCIGKDKGKSVSEKVDALTKIITGMNYDQFDKKRSVIEDTGR ENAEREKFKKIISLYLTVIYHILKNIVNINARYVIGFHCVERDAQLYKEKGYDINLKKLEE KGFSSVTKLCAGIDETAPDKRKDVEKEMAERAKESIDSLESANPKLYANYIKYSDEKKA EEFTRQINREKAKTALNAYLRNTKWNVIIREDLLRIDNKTCTLFRNKAVHLEVARYVH AYINDIAEVNSYFQLYHYIMQRIIMNERYEKSSGKVSEYFDAVNDEKKYNDRLLKLLC VPFGYCIPRFKNLSIEALFDRNEAAKFDKEKKKVSGNSGSTPPKKKRKVEDGEGPAAK RVKLDSGAAPAAKKKKLDYKDDDDKLQLPPLERLTL |
| V3 (Cas13d-NCS) | MIEKKKSFAKGMGVKSTLVSGSKVYMTTFAEGSDARLEKIVEGDSIRSVNEGEAFSA EMADKNAGYKIGNAKFSHPKGYAVVANNPLYTGPVQQDMLGLKETLEKRYFGESA DGNDNICIQVIHNILDIEKILAEYITNAAYAVNNISGLDKDIIGFGKFSTVYTYDEFKDPE HHRAAFNNNDKLINAIKAQYDEFDNFLDNPRLGYFGQAFFSKEGRNYIINYGNECYD ILALLSGLRHWVVHNNEEESRISRTWLYNLDKNLDNEYISTLNYLYDRITNELTNSFSK NSAANVNYIAETLGINPAEFAEQYFRFSIMKEQKNLGFNITKLREVMLDRKDMSEIR KNHKVFDSIRTKVYTMMDFVIYRYYIEEDAKVAAANKSLPDNEKSLSEKDIFVINLRG SFNDDQKDALYYDEANRIWRKLENIMHNIKEFRGNKTREYKKKDAPRLPRILPAGRD VSAFSKLMYALTMFLDGKEINDLLTTLINKFDNIQSFLKVMPLIGVNAKFVEEYAFFK DSAKIADELRLIKSFARMGEPIADARRAMYIDAIRILGTNLSYDELKALADTFSLDENG NKLKKGKHGMRNFIINNVISNKRFHYLIRYGDPAHLHEIAKNEAVVKFVLGRIADIQK KQGQNGKNQIDRYYETCIGKDKGKSVSEKVDALTKIITGMNYDQFDKKRSVIEDTGR ENAEREKFKKIISLYLTVIYHILKNIVNINARYVIGFHCVERDAQLYKEKGYDINLKKLEE KGFSSVTKLCAGIDETAPDKRKDVEKEMAERAKESIDSLESANPKLYANYIKYSDEKKA EEFTRQINREKAKTALNAYLRNTKWNVIIREDLLRIDNKTCTLFRNKAVHLEVARYVH AYINDIAEVNSYFQLYHYIMQRIIMNERYEKSSGKVSEYFDAVNDEKKYNDRLLKLLC VPFGYCIPRFKNLSIEALFDRNEAAKFDKEKKKVSGNSGSTPPKKKRKVEDGEGLQLP PLERLTLSGAAPAAKKKKLDYKDDDDK |
| V4 | MIEKKKSFAKGMGVKSTLVSGSKVYMTTFAEGSDARLEKIVEGDSIRSVNEGEAFSA EMADKNAGYKIGNAKFSHPKGYAVVANNPLYTGPVQQDMLGLKETLEKRYFGESA DGNDNICIQVIHNILDIEKILAEYITNAAYAVNNISGLDKDIIGFGKFSTVYTYDEFKDPE HHRAAFNNNDKLINAIKAQYDEFDNFLDNPRLGYFGQAFFSKEGRNYIINYGNECYD ILALLSGLRHWVVHNNEEESRISRTWLYNLDKNLDNEYISTLNYLYDRITNELTNSFSK |

| | |
|---|---|
| | NSAANVNYIAETLGINPAEFAEQYFRFSIMKEQKNLGFNITKLREVMLDRKDMSEIRKNHKVFDSIRTKVYTMMDFVIYRYYIEEDAKVAAANKSLPDNEKSLSEKDIFVINLRGSFNDDQKDALYYDEANRIWRKLENIMHNIKEFRGNKTREYKKKDAPRLPRILPAGRDVSAFSKLMYALTMFLDGKEINDLLTTLINKFDNIQSFLKVMPLIGVNAKFVEEYAFFKDSAKIADELRLIKSFARMGEPIADARRAMYIDAIRILGTNLSYDELKALADTFSLDENGNKLKKGKHGMRNFIINNVISNKRFHYLIRYGDPAHLHEIAKNEAVVKFVLGRIADIQKKQGQNGKNQIDRYYETCIGKDKGKSVSEKVDALTKIITGMNYDQFDKKRSVIEDTGRENAEREKFKKIISLYLTVIYHILKNIVNINARYVIGFHCVERDAQLYKEKGYDINLKKLEEKGFSSVTKLCAGIDETAPDKRKDVEKEMAERAKESIDSLESANPKLYANYIKYSDEKKAEEFTRQINREKAKTALNAYLRNTKWNVIIREDLLRIDNKTCTLFRNKAVHLEVARYVHAYINDIAEVNSYFQLYHYIMQRIIMNERYEKSSGKVSEYFDAVNDEKKYNDRLLKLLCVPFGYCIPRFKNLSIEALFDRNEAAKFDKEKKKVSGNSGSTPPKKKRKVEDGEGLQLPPLERLTLSGAAPAAKKKKLDYKDDDDKLQLPPLERLTL |
| V5 (Cytosolic Cas13d) | MIEKKKSFAKGMGVKSTLVSGSKVYMTTFAEGSDARLEKIVEGDSIRSVNEGEAFSAEMADKNAGYKIGNAKFSHPKGYAVVANNPLYTGPVQQDMLGLKETLEKRYFGESADGNDNICIQVIHNILDIEKILAEYITNAAYAVNNISGLDKDIIGFGKFSTVYTYDEFKDPEHHRAAFNNNDKLINAIKAQYDEFDNFLDNPRLGYFGQAFFSKEGRNYIINYGNECYDILALLSGLRHWVVHNNEEESRISRTWLYNLDKNLDNEYISTLNYLYDRITNELTNSFSKNSAANVNYIAETLGINPAEFAEQYFRFSIMKEQKNLGFNITKLREVMLDRKDMSEIRKNHKVFDSIRTKVYTMMDFVIYRYYIEEDAKVAAANKSLPDNEKSLSEKDIFVINLRGSFNDDQKDALYYDEANRIWRKLENIMHNIKEFRGNKTREYKKKDAPRLPRILPAGRDVSAFSKLMYALTMFLDGKEINDLLTTLINKFDNIQSFLKVMPLIGVNAKFVEEYAFFKDSAKIADELRLIKSFARMGEPIADARRAMYIDAIRILGTNLSYDELKALADTFSLDENGNKLKKGKHGMRNFIINNVISNKRFHYLIRYGDPAHLHEIAKNEAVVKFVLGRIADIQKKQGQNGKNQIDRYYETCIGKDKGKSVSEKVDALTKIITGMNYDQFDKKRSVIEDTGRENAEREKFKKIISLYLTVIYHILKNIVNINARYVIGFHCVERDAQLYKEKGYDINLKKLEEKGFSSVTKLCAGIDETAPDKRKDVEKEMAERAKESIDSLESANPKLYANYIKYSDEKKAEEFTRQINREKAKTALNAYLRNTKWNVIIREDLLRIDNKTCTLFRNKAVHLEVARYVHAYINDIAEVNSYFQLYHYIMQRIIMNERYEKSSGKVSEYFDAVNDEKKYNDRLLKLLCVPFGYCIPRFKNLSIEALFDRNEAAKFDKEKKKVSGNSGSLQLPPLERLTLGSGDYKDDDDK |

**Supplementary Table 3 |** Other DNA and protein sequences used in this study

| | |
|---|---|
| Nanoluciferase-PEST reporter | ATGGTCTTTACACTGGAAGATTTCGTCGGCGACTGGCGGCAGACAGCTGGCTACAATCTGGACCAGGTGCTGGAACAAGGCGGCGTGTCCTCTCTGTTTCAGAACCTGGGAGTGTCTGTGACCCCTATCCAGAGAATCGTGCTGAGCGGCGAGAACGGCCTGAAGATCGACATCCACGTGATCATCCCTTACGAGGGCCTGTCCGGCGATCAGATGGGACAGATCGAGAAGATCTTTAAGGTGGTGTACCCCGTGGACGACCACCACTTCAAAGTGATCCTGCACTACGGCACCCTGGTCATCGATGGCGTGACCCCAAACATGATCGACTACTTCGGCAGACCCTACGAGGGAATCGCCGTGTTCGACGGCAAGAAAATCACCGTGACCGGCACACTGTGGAACGGCAACAAGATCATCGACGAGAGACTGATCAACCCCGACGGCAGCCTGCTGTTCAGAGTGACAATCAACGGCGTGACAGGCTGGCGGCTGTGCGAAAGAATCCTTGCTGGCAGCGGAAACAGCCACGGCTTCCCACCCGAGGTGGAAGAACAGGCTGCCGGAACCCTGCCTATGAGCTGCGCTCAGGAATCCGGCATGGACAGGCACCCTGCCGCTTGTGCCTCCGCCAGAATCAACGTGTAG |

| | |
|---|---|
| mRuby3 | ATGGTGTCCAAGGGCGAAGAACTGATCAAAGAAAACATGCGGATGAAGGTGGTC<br>ATGGAAGGCAGCGTGAACGGCCACCAGTTCAAGTGTACAGGCGAAGGCGAGGGC<br>AGACCTTATGAGGGCGTGCAGACCATGAGAATCAAAGTGATCGAAGGCGGCCCTC<br>TGCCTTTCGCTTTCGACATCCTGGCCACCAGCTTTATGTACGGCAGCAGAACCTTCA<br>TCAAGTACCCCGCTGACATCCCCGATTTCTTCAAGCAGAGCTTCCCCGAGGGCTTCA<br>CCTGGGAGAGAGTGACCAGATACGAGGATGGCGGCGTGGTCACAGTGACCCAGG<br>ACACATCTCTGGAAGATGGCGAGCTGGTGTACAACGTGAAAGTGCGGGGCGTGA<br>ACTTCCCCTCTAACGGCCCTGTGATGCAGAAAAAGACCAAAGGCTGGGAGCCCAA<br>CACCGAGATGATGTACCCTGCTGATGGCGGCCTGAGAGGCTACACAGATATCGCC<br>CTGAAAGTGGACGGCGGAGGCCATCTGCACTGCAACTTCGTGACCACCTACAGGT<br>CCAAGAAAACCGTGGGCAACATCAAGATGCCTGGGGTGCACGCCGTGGACCACAG<br>ACTGGAAAGAATCGAGGAAAGCGACAACGAGACATACGTGGTGCAGCGCGAAGT<br>GGCCGTGGCCAAGTACTCTAATCTCGGAGGCGGCATGGACGAGCTGTACAAA |
| VEE replicon (B18R, E3L, mGreenLantern insert) | ATGGGCGGCGCATGAGAGAAGCCCAGACCAATTACCTACCCAAAATGGAGAAAGT<br>TCACGTTGACATCGAGGAAGACAGCCCATTCCTCAGAGCTTTGCAGCGGAGCTTCC<br>CGCAGTTTGAGGTAGAAGCCAAGCAGGTCACTGATAATGACCATGCTAATGCCAG<br>AGCGTTTTCGCATCTGGCTTCAAAACTGATCGAAACGGAGGTGGACCCATCCGACA<br>CGATCCTTGACATTGGAAGTGCGCCCGCCCGCAGAATGTATTCTAAGCACAAGTAT<br>CATTGTATCTGTCCGATGAGATGTGCGGAAGATCCGGACAGATTGTATAAGTATGC<br>AACTAAGCTGAAGAAAAACTGTAAGGAAATAACTGATAAGGAATTGGACAAGAAA<br>ATGAAGGAGCTGGCCGCCGTCATGAGCGACCCTGACCTGGAAACTGAGACTATGT<br>GCCTCCACGACGACGAGTCGTGTCGCTACGAAGGGCAAGTCGCTGTTTACCAGGA<br>TGTATACGCGGTTGACGGACCGACAAGTCTCTATCACCAAGCCAATAAGGGAGTTA<br>GAGTCGCCTACTGGATAGGCTTTGACACCACCCCTTTTATGTTTAAGAACTTGGCTG<br>GAGCATATCCATCATACTCTACCAACTGGGCCGACGAAACCGTGTTAACGGCTCGT<br>AACATAGGCCTATGCAGCTCTGACGTTATGGAGCGGTCACGTAGAGGGATGTCCA<br>TTCTTAGAAAGAAGTATTTGAAACCATCCAACAATGTTCTATTCTCTGTTGGCTCGA<br>CCATCTACCACGAGAAGAGGGACTTACTGAGGAGCTGGCACCTGCCGTCTGTATTT<br>CACTTACGTGGCAAGCAAAATTACACATGTCGGTGTGAGACTATAGTTAGTTGCGA<br>CGGGTACGTCGTTAAAAGAATAGCTATCAGTCCAGGCCTGTATGGGAAGCCTTCA<br>GGCTATGCTGCTACGATGCACCGCGAGGGATTCTTGTGCTGCAAAGTGACAGACA<br>CATTGAACGGGGAGAGGGTCTCTTTTCCCGTGTGCACGTATGTGCCAGCTACATTG<br>TGTGACCAAATGACTGGCATACTGGCAACAGATGTCAGTGCGGACGACGCGCAAA<br>AACTGCTGGTTGGGCTCAACCAGCGTATAGTCGTCAACGGTCGCACCCAGAGAAA<br>CACCAATACCATGAAAAATTACCTTTTGCCCGTAGTGGCCCAGGCATTTGCTAGGT<br>GGGCAAAGGAATATAAGGAAGATCAAGAAGATGAAAGGCCACTAGGACTACGAG<br>ATAGACAGTTAGTCATGGGGTGTTGTTGGGCTTTTAGAAGGCACAAGATAACATCT<br>ATTTATAAGCGCCCGGATACCCAAACCATCATCAAAGTGAACAGCGATTTCCACTC<br>ATTCGTGCTGCCCAGGATAGGCAGTAACACATTGGAGATCGGGCTGAGAACAAGA<br>ATCAGGAAAATGTTAGAGGAGCACAAGGAGCCGTCACCTCTCATTACCGCCGAGG<br>ACGTACAAGAAGCTAAGTGCGCAGCCGATGAGGCTAAGGAGGTGCGTGAAGCCG<br>AGGAGTTGCGCGCAGCTCTACCACCTTTGGCAGCTGATGTTGAGGAGCCCACTCTG<br>GAGGCAGACGTCGACTTGATGTTACAAGAGGCTGGGGCCGGCTCAGTGGAGACA<br>CCTCGTGGCTTGATAAAGGTTACCAGCTACGATGGCGAGGACAAGATCGGCTCTTA<br>CGCTGTGCTTTCTCCGCAGGCTGTACTCAAGAGTGAAAAATTATCTTGCATCCACCC<br>TCTCGCTGAACAAGTCATAGTGATAACACACTCTGGCCGAAAAGGGCGTTATGCCG |

TGGAACCATACCATGGTAAAGTAGTGGTGCCAGAGGGACATGCAATACCCGTCCA
GGACTTTCAAGCTCTGAGTGAAAGTGCCACCATTGTGTACAACGAACGTGAGTTCG
TAAACAGGTACCTGCACCATATTGCCACACATGGAGGAGCGCTGAACACTGATGA
AGAATATTACAAAACTGTCAAGCCCAGCGAGCACGACGGCGAATACCTGTACGAC
ATCGACAGGAAACAGTGCGTCAAGAAAGAACTAGTCACTGGGCTAGGGCTCACAG
GCGAGCTGGTGGATCCTCCCTTCCATGAATTCGCCTACGAGAGTCTGAGAACACGA
CCAGCCGCTCCTTACCAAGTACCAACCATAGGGGTGTATGGCGTGCCAGGATCAG
GCAAGTCTGGCATCATTAAAAGCGCAGTCACCAAAAAAGATCTAGTGGTGAGCGC
CAAGAAAGAAAACTGTGCAGAAATTATAAGGGACGTCAAGAAAATGAAAGGGCT
GGACGTCAATGCCAGAACTGTGGACTCAGTGCTCTTGAATGGATGCAAACACCCC
GTAGAGACCCTGTATATTGACGAAGCTTTTGCTTGTCATGCAGGTACTCTCAGAGC
GCTCATAGCCATTATAAGACCTAAAAAGGCAGTGCTCTGCGGGGATCCCAAACAGT
GCGGTTTTTTTAACATGATGTGCCTGAAAGTGCATTTTAACCACGAGATTTGCACAC
AAGTCTTCCACAAAAGCATCTCTCGCCGTTGCACTAAATCTGTGACTTCGGTCGTCT
CAACCTTGTTTTACGACAAAAAAATGAGAACGACGAATCCGAAAGAGACTAAGATT
GTGATTGACACTACCGGCAGTACCAAACCTAAGCAGGACGATCTCATTCTCACTTG
TTTCAGAGGGTGGGTGAAGCAGTTGCAAATAGATTACAAAGGCAACGAAATAATG
ACGGCAGCTGCCTCTCAAGGGCTGACCCGTAAAGGTGTGTATGCCGTTCGGTACA
AGGTGAATGAAAATCCTCTGTACGCACCCACCTCAGAACATGTGAACGTCCTACTG
ACCCGCACGGAGGACCGCATCGTGTGGAAAACACTAGCCGGCGACCCATGGATAA
AAACACTGACTGCCAAGTACCCTGGGAATTTCACTGCCACGATAGAGGAGTGGCA
AGCAGAGCATGATGCCATCATGAGGCACATCTTGGAGAGACCGGACCCTACCGAC
GTCTTCCAGAATAAGGCAAACGTGTGTTGGGCCAAGGCTTTAGTGCCGGTGCTGA
AGACCGCTGGCATAGACATGACCACTGAACAATGGAACACTGTGGATTATTTTGAA
ACGGACAAAGCTCACTCAGCAGAGATAGTATTGAACCAACTATGCGTGAGGTTCTT
TGGACTCGATCTGGACTCCGGTCTATTTTCTGCACCCACTGTTCCGTTATCCATTAG
GAATAATCACTGGGATAACTCCCCGTCGCCTAACATGTACGGGCTGAATAAAGAAG
TGGTCCGTCAGCTCTCTCGCAGGTACCCACAACTGCCTCGGGCAGTTGCCACTGGA
AGAGTCTATGACATGAACACTGGTACACTGCGCAATTATGATCCGCGCATAAACCT
AGTACCTGTAAACAGAAGACTGCCTCATGCTTTAGTCCTCCACCATAATGAACACCC
ACAGAGTGACTTTTCTTCATTCGTCAGCAAATTGAAGGGCAGAACTGTCCTGGTGG
TCGGGGAAAAGTTGTCCGTCCCAGGCAAAATGGTTGACTGGTTGTCAGACCGGCC
TGAGGCTACCTTCAGAGCTCGGCTGGATTTAGGCATCCCAGGTGATGTGCCCAAAT
ATGACATAATATTTGTTAATGTGAGGACCCCATATAAATACCATCACTATCAGCAGT
GTGAAGACCATGCCATTAAGCTTAGCATGTTGACCAAGAAAGCTTGTCTGCATCTG
AATCCCGGCGGAACCTGTGTCAGCATAGGTTATGGTTACGCTGACAGGGCCAGCG
AAAGCATCATTGGTGCTATAGCGCGGCAGTTCAAGTTTTCCCGGGTATGCAAACCG
AAATCCTCACTTGAAGAGACGGAAGTTCTGTTTGTATTCATTGGGTACGATCGCAA
GGCCCGTACGCACAATTCTTACAAGCTTTCATCAACCTTGACCAACATTTATACAGG
TTCCAGACTCCACGAAGCCGGATGTGCACCCTCATATCATGTGGTGCGAGGGGATA
TTGCCACGGCCACCGAAGGAGTGATTATAAATGCTGCTAACAGCAAAGGACAACC
TGGCGGAGGGGTGTGCGGAGCGCTGTATAAGAAATTCCCGGAAAGCTTCGATTTA
CAGCCGATCGAAGTAGGAAAAGCGCGACTGGTCAAAGGTGCAGCTAAACATATCA
TTCATGCCGTAGGACCAAACTTCAACAAAGTTTCGGAGGTTGAAGGTGACAAACA
GTTGGCAGAGGCTTATGAGTCCATCGCTAAGATTGTCAACGATAACAATTACAAGT
CAGTAGCGATTCCACTGTTGTCCACCGGCATCTTTTCCGGGAACAAAGATCGACTA

| | |
|---|---|
| | ACCCAATCATTGAACCATTTGCTGACAGCTTTAGACACCACTGATGCAGATGTAGC<br>CATATACTGCAGGGACAAGAAATGGGAAATGACTCTCAAGGAAGCAGTGGCTAGG<br>AGAGAAGCAGTGGAGGAGATATGCATATCCGACGACTCTTCAGTGACAGAACCTG<br>ATGCAGAGCTGGTGAGGGTGCATCCGAAGAGTTCTTTGGCTGGAAGGAAGGGCT<br>ACAGCACAAGCGATGGCAAAACTTTCTCATATTTGGAAGGGACCAAGTTTCACCAG<br>GCGGCCAAGGATATAGCAGAAATTAATGCCATGTGGCCCGTTGCAACGGAGGCCA<br>ATGAGCAGGTATGCATGTATATCCTCGGAGAAAGCATGAGCAGTATTAGGTCGAA<br>ATGCCCCGTCGAAGAGTCGGAAGCCTCCACACCACCTAGCACGCTGCCTTGCTTGT<br>GCATCCATGCCATGACTCCAGAAAGAGTACAGCGCCTAAAAGCCTCACGTCCAGAA<br>CAAATTACTGTGTGCTCATCCTTTCCATTGCCGAAGTATAGAATCACTGGTGTGCAG<br>AAGATCCAATGCTCCCAGCCTATATTGTTCTCACCGAAAGTGCCTGCGTATATTCAT<br>CCAAGGAAGTATCTCGTGGAAACACCACCGGTAGACGAGACTCCGGAGCCATCGG<br>CAGAGAACCAATCCACAGAGGGGACACCTGAACAACCACCACTTATAACCGAGGA<br>TGAGACCAGGACTAGAACGCCTGAGCCGATCATCATCGAAGAGGAAGAAGAGGA<br>TAGCATAAGTTTGCTGTCAGATGGCCCGACCCACCAGGTGCTGCAAGTCGAGGCA<br>GACATTCACGGGCCGCCCTCTGTATCTAGCTCATCCTGGTCCATTCCTCATGCATCC<br>GACTTTGATGTGGACAGTTTATCCATACTTGACACCCTGGAGGGAGCTAGCGTGAC<br>CAGCGGGGCAACGTCAGCCGAGACTAACTCTTACTTCGCAAAGAGTATGGAGTTTC<br>TGGCGCGACCGGTGCCTGCGCCTCGAACAGTATTCAGGAACCCTCCACATCCCGCT<br>CCGCGCACAAGAACACCGTCACTTGCACCCAGCAGGGCCTGCTCGAGAACCAGCCT<br>AGTTTCCACCCCGCCAGGCGTGAATAGGGTGATCACTAGAGAGGAGCTCGAGGCG<br>CTTACCCCGTCACGCACTCCTAGCAGGTCGGTCTCGAGAACCAGCCTGGTCTCCAA<br>CCCGCCAGGCGTAAATAGGGTGATTACAAGAGAGGAGTTTGAGGCGTTCGTAGCA<br>CAACAACAATGACGGTTTGATGCGGGTGCATACATCTTTTCCTCCGACACCGGTCA<br>AGGGCATTTACAACAAAAATCAGTAAGGCAAACGGTGCTATCCGAAGTGGTGTTG<br>GAGAGGACCGAATTGGAGATTTCGTATGCCCCGCGCCTCGACCAAGAAAAAGAAG<br>AATTACTACGCAAGAAATTACAGTTAAATCCCACACCTGCTAACAGAAGCAGATAC<br>CAGTCCAGGAAGGTGGAGAACATGAAAGCCATAACAGCTAGACGTATTCTGCAAG<br>GCCTAGGGCATTATTTGAAGGCAGAAGGAAAAGTGGAGTGCTACCGAACCCTGCA<br>TCCTGTTCCTTTGTATTCATCTAGTGTGAACCGTGCCTTTTCAAGCCCCAAGGTCGC<br>AGTGGAAGCCTGTAACGCCATGTTGAAAGAGAACTTTCCGACTGTGGCTTCTTACT<br>GTATTATTCCAGAGTACGATGCCTATTTGGACATGGTTGACGGAGCTTCATGCTGC<br>TTAGACACTGCCAGTTTTTGCCCTGCAAAGCTGCGCAGCTTTCCAAAGAAACACTCC<br>TATTTGGAACCCACAATACGATCGGCAGTGCCTTCAGCGATCCAGAACACGCTCCA<br>GAACGTCCTGGCAGCTGCCACAAAAAGAAATTGCAATGTCACGCAAATGAGAGAA<br>TTGCCCGTATTGGATTCGGCGGCCTTTAATGTGGAATGCTTCAAGAAATATGCGTG<br>TAATAATGAATATTGGGAAACGTTTAAAGAAAACCCCATCAGGCTTACTGAAGAAA<br>ACGTGGTAAATTACATTACCAAATTAAAAGGACCAAAAGCTGCTGCTCTTTTTGCG<br>AAGACACATAATTTGAATATGTTGCAGGACATACCAATGGACAGGTTTGTAATGGA<br>CTTAAAGAGAGACGTGAAAGTGACTCCAGGAACAAAACATACTGAAGAACGGCCC<br>AAGGTACAGGTGATCCAGGCTGCCGATCCGCTAGCAACAGCGTATCTGTGCGGAA<br>TCCACCGAGAGCTGGTTAGGAGATTAAATGCGGTCCTGCTTCCGAACATTCATACA<br>CTGTTTGATATGTCGGCTGAAGACTTTGACGCTATTATAGCCGAGCACTTCCAGCCT<br>GGGGATTGTGTTCTGGAAACTGACATCGCGTCGTTTGATAAAAGTGAGGACGACG<br>CCATGGCTCTGACCGCGTTAATGATTCTGGAAGACTTAGGTGTGGACGCAGAGCT<br>GTTGACGCTGATTGAGGCGGCTTTCGGCGAAATTTCATCAATACATTTGCCCACTA |

| | |
|---|---|
| | AAACTAAATTTAAATTCGGAGCCATGATGAAATCTGGAATGTTCCTCACACTGTTTG<br>TGAACACAGTCATTAACATTGTAATCGCAAGCAGAGTGTTGAGAGAACGGCTAACC<br>GGATCACCATGTGCAGCATTCATTGGAGATGACAATATCGTGAAAGGAGTCAAATC<br>GGACAAATTAATGGCAGACAGGTGCGCCACCTGGTTGAATATGGAAGTCAAGATT<br>ATAGATGCTGTGGTGGGCGAGAAAGCGCCTTATTTCTGTGGAGGGTTTATTTTGTG<br>TGACTCCGTGACCGGCACAGCGTGCCGTGTGGCAGACCCCCTAAAAAGGCTGTTTA<br>AGCTTGGCAAACCTCTGGCAGCAGACGATGAACATGATGATGACAGGAGAAGGG<br>CATTGCATGAAGAGTCAACACGCTGGAACCGAGTGGGTATTCTTTCAGAGCTGTGC<br>AAGGCAGTAGAATCAAGGTATGAAACCGTAGGAACTTCCATCATAGTTATGGCCAT<br>GACTACTCTAGCTAGCAGTGTTAAATCATTCAGCTACCTGAGAGGGGCCCCTATAA<br>CTCTCTACGGCTAACCTGAATGGACTACGACATAGTCTAGTCCGCCAAGTCTAGCA<br>TATGGCCGCCACCATGGGCACCATGAAGATGATGGTGCACATCTACTTCGTGTCCC<br>TGCTGCTGCTCCTGTTCCACAGCTACGCCATCGACATCGAGAACGAGATCACCGAG<br>TTCTTCAACAAGATGCGGGACACCCTGCCTGCCAAGGACAGCAAGTGGCTGAATCC<br>CGCCTGTATGTTCGGCGGCACAATGAACGATATCGCCGCTCTGGGCGAGCCCTTCA<br>GCGCTAAGTGTCCTCCTATCGAGGACAGCCTGCTGAGCCACCGGTACAAGGACTAC<br>GTGGTCAAGTGGGAGAGACTCGAGAAGAACCGGCGGAGACAGGTGTCCAACAAG<br>AGAGTGAAGCACGGCGACCTGTGGATCGCCAACTACACCAGCAAGTTCAGCAATC<br>GGAGATACCTGTGCACCGTGACCACCAAGAACGGCGATTGTGTGCAGGGAATCGT<br>GCGGAGCCACATCAGAAAGCCTCCAAGCTGCATCCCCAAGACCTACGAGCTGGGC<br>ACCCACGATAAGTACGGCATCGATCTGTACTGCGGCATCCTGTACGCCAAGCACTA<br>CAACAACATCACCTGGTATAAGGACAACAAAGAGATCAACATCGACGACATCAAG<br>TACAGCCAGACCGGCAAAGAGCTGATCATTCACAACCCCGAGCTGGAAGATAGCG<br>GCAGATACGACTGCTACGTGCACTACGACGACGTGCGGATCAAGAACGACATCGT<br>GGTGTCCCGGTGCAAGATCCTGACAGTGATCCCCAGCCAGGACCACCGGTTCAAG<br>CTGATTCTGGACCCCAAGATCAACGTGACCATCGGCGAGCCCGCCAACATCACATG<br>TACCGCCGTGTCTACCTCTCTGCTGATCGACGATGTGCTGATTGAGTGGGAGAACC<br>CCAGCGGCTGGCTGATCGGCTTCGACTTCGATGTGTACAGCGTGCTGACAAGCAG<br>AGGCGGCATCACAGAGGCCACACTGTACTTCGAGAACGTGACCGAAGAGTACATC<br>GGCAACACCTACAAGTGCAGGGGCCACAACTACTACTTCGAAAAGACCCTGACCAC<br>CACCGTGGTGCTGGAAGGATCTGGCGCCACCAACTTCAGTCTGCTGAAACAGGCT<br>GGCGACGTGGAAGAGAACCCCGGACCTAGCAAGATCTACATCGACGAGCGGAGC<br>AACGCCGAGATTGTGTGCGAGGCCATCAAGACCATCGGAATCGAAGGCGCCACAG<br>CCGCTCAGCTGACCAGACAGCTGAACATGGAAAAGCGGGAAGTGAACAAGGCCCT<br>GTACGACCTGCAGAGAAGCGCCATGGTGTACAGCAGCGACGACATCCCTCCTCGG<br>TGGTTTATGACCACAGAGGCCGACAAGCCTGACGCCGATGCTATGGCCGACGTGA<br>TCATCGACGACGTGTCCCGCGAGAAGTCCATGAGAGAGGACCACAAGAGCTTCGA<br>CGATGTGATCCCCGCCAAGAAGATCATCGATTGGAAGGGCGCCAATCCTGTGACC<br>GTGATCAACGAGTACTGCCAGATCACCAGAAGAGACTGGTCCTTCCGGATCGAGA<br>GCGTGGGCCCTAGCAATAGCCCTACCTTCTACGCCTGCGTGGACATCGACGGCAGA<br>GTGTTCGATAAGGCCGACGGCAAGAGCAAGCGGGACGCCAAAAACAATGCCGCC<br>AAGCTGGCCGTGGATAAGCTGCTGGGCTATGTGATCATCCGGTTCGGCAGCGGGG<br>CGACGAACTTCAGCCTGCTCAAGCAGGCCGGGGATGTCGAGGAAAACCCCGGCC<br>CGCCGGCGTGTCCAAAGGGGAAGAGCTGTTTACTGGGGTAGTGCCAATACTTGTT<br>GAATTGGATGGGGATGTTAATGGGCATAAATTTTCTGTTAGAGGTGAAGGTGAAG<br>GAGACGCTACGAATGGAAAATTGACTCTTAAATTTATTTGTACGACAGGAAAACTC |

| | |
|---|---|
| | CCTGTTCCTTGGCCTACTCTTGTTACGACTCTGGGGTATGGTGTCGCATGTTTTGCT<br>AGGTATCCAGATCACATGAAACAACATGATTTCTTTAAATCAGCTATGCCAGAGGG<br>TTATGTGCAAGAACGTACGATTTCATTTAAAGACGACGGAACTTATAAAACAAGGG<br>CTGAGGTCAAATTTGAAGGAGATACTCTCGTTAATAGGATAGTCTTGAAAGGAATT<br>GACTTCAAAGAGGACGGGAACATTCTCGGACACAAGCTCGAATATAATTTTAACTC<br>TCATAAAGTGTACATAACCGCCGATAAACAAAAGAATGGTATTAAGGCGAATTTTA<br>AAACACGGCATAATGTCGAAGATGGTGGAGTCCAACTGGCAGATCATTATCAACA<br>AAATACACCAATTGGGGATGGACCAGTTTTGTTGCCTGATAATCATTATTTGTCCCA<br>CCAAAGTAAGTTGTCAAAGGACCCGAATGAAAAGAGGGACCATATGGTGCTCAAA<br>GAACGTGTCACAGCTGCAGGCATCACTCACGATATGGATGAACTTTATAAATGAGC<br>GGCCGCCGTTGTACACCCCCCTCTCCCTCCCCCCCCCCTAACGTTACTGGCCGAAGC<br>CGCTTGGAATAAGGCCGGTGTGCGTTTGTCTATATGTTATTTTCCACCATATTGCCG<br>TCTTTTGGCAATGTGAGGGCCCGGAAACCTGGCCCTGTCTTCTTGACGAGCATTCCT<br>AGGGGTCTTTCCCCTCTCGCCAAAGGAATGCAAGGTCTGTTGAATGTCGTGAAGGA<br>AGCAGTTCCTCTGGAAGCTTCTTGAAGACAAACAACGTCTGTAGCGACCCTTTGCA<br>GGCAGCGGAACCCCCCACCTGGCGACAGGTGCCTCTGCGGCCAAAAGCCACGTGT<br>ATAAGATACACCTGCAAAGGCGGCACAACCCCAGTGCCACGTTGTGAGTTGGATA<br>GTTGTGGAAAGAGTCAAATGGCTCTCCTCAAGCGTATTCAACAAGGGGCTGAAGG<br>ATGCCCAGAAGGTACCCCATTGTATGGGATCTGATCTGGGGCCTCGGTGCACATGC<br>TTTACATGTGTTTAGTCGAGGTTAAAAAACGTCTAGGCCCCCCGAACCACGGGGAC<br>GTGGTTTTCCTTTGAAAAACACGATGATAATATGGCCACCACCATGACCGAGTACA<br>AGCCCACGGTGCGCCTCGCCACCCGCGACGACGTCCCCAGGGCCGTACGCACCCTC<br>GCCGCCGCGTTCGCCGACTACCCCGCCACGCGCCACACCGTCGATCCGGACCGCCA<br>CATCGAGCGGGTCACCGAGCTGCAAGAACTCTTCCTCACGCGCGTCGGGCTCGAC<br>ATCGGCAAGGTGTGGGTCGCGGACGACGGCGCCGCGGTGGCGGTCTGGACCACG<br>CCGGAGAGCGTCGAAGCGGGGGCGGTGTTCGCCGAGATCGGCCCGCGCATGGCC<br>GAGTTGAGCGGTTCCCGGCTGGCCGCGCAGCAACAGATGGAAGGCCTCCTGGCGC<br>CGCACCGGCCCAAGGAGCCCGCGTGGTTCCTGGCCACCGTCGGCGTCTCGCCCGA<br>CCACCAGGGCAAGGGTCTGGGCAGCGCCGTCGTGCTCCCCGGAGTGGAGGCGGC<br>CGAGCGCGCCGGGGTGCCCGCCTTCCTGGAGACCTCCGCGCCCCGCAACCTCCCCT<br>TCTACGAGCGGCTCGGCTTCACCGTCACCGCCGACGTCGAGGTGCCCGAAGGACC<br>GCGCACCTGGTGCATGACCCGCAAGCCCGGTGCCTGAGAATTGGCAAGCTGCTTA<br>CATAGAACTCGCGGCGATTGGCATGCCGCCTTAAAATTTTTATTTTATTTTTTCTTTT<br>CTTTTCCGAATCGGATTTTGTTTTTAATATTTCAAAAAAAAAAAAAAAAAAAAAAAA<br>AA… |
| Cas13d-NCS-P2A-mRuby3 mRNA (with 5’, 3’ UTR) | AATACAAGCTACTTGTTCTTTTTGCATTGTACAACTCACTATTTGTTTTCGCGCCCAG<br>TTGCAAAAAGTGTCGCCACCATGATCGAGAAGAAGAAGTCCTTCGCCAAAGGCAT<br>GGGCGTGAAGTCGACCCTGGTGTCCGGCAGCAAGGTGTACATGACCACATTCGCC<br>GAGGGCAGCGACGCCAGACTGGAAAAGATTGTGGAAGGCGACAGCATCCGCAGC<br>GTGAACGAGGGCGAAGCTTTCTCTGCCGAGATGGCCGATAAGAACGCTGGCTACA<br>AGATCGGCAACGCCAAGTTCTCTCACCCCAAGGGCTATGCCGTGGTGGCTAACAAC<br>CCACTGTACACAGGACCCGTGCAGCAGGATATGCTGGGCCTGAAAGAGACTCTGG<br>AAAAGCGGTACTTCGGCGAGAGCGCCGACGGCAACGACAACATCTGCATCCAAGT<br>GATCCACAACATCCTGGACATCGAAAAGATCCTGGCCGAGTACATCACCAACGCCG<br>CCTACGCCGTGAACAACATCAGCGGCCTGGACAAGGACATCATCGGCTTCGGCAA<br>GTTCAGCACCGTGTACACCTACGACGAGTTCAAGGACCCCGAGCACCACAGAGCC |

| | |
|---|---|
| | GCCTTCAACAACAACGACAAGCTGATCAACGCCATCAAGGCCCAGTACGATGAGTT<br>CGACAACTTCCTGGATAACCCCAGACTGGGCTACTTCGGCCAAGCCTTCTTTAGCA<br>AAGAGGGCCGCAACTACATCATCAACTACGGGAACGAGTGCTACGACATTCTGGC<br>CCTCTTAAGCGGCCTGAGGCACTGGGTCGTGCACAACAATGAGGAAGAGAGCCGG<br>ATATCCAGAACCTGGCTGTACAACCTGGACAAAAATCTGGACAACGAGTATATCAG<br>CACCCTGAACTACCTGTACGACCGGATCACAAACGAGCTGACCAACAGCTTCAGCA<br>AGAACAGCGCCGCCAACGTGAACTATATCGCCGAGACACTGGGCATCAACCCCGC<br>CGAGTTTGCCGAGCAGTACTTCAGATTCAGCATCATGAAGGAACAGAAGAACCTG<br>GGCTTCAACATCACAAAGCTGCGGGAAGTGATGCTGGACAGAAAGGACATGAGC<br>GAGATCCGCAAGAACCACAAGGTGTTCGACTCCATCAGGACCAAGGTCTATACCAT<br>GATGGACTTCGTGATCTATCGGTACTACATCGAAGAGGACGCCAAGGTGGCCGCT<br>GCCAACAAGTCTCTGCCCGACAACGAGAAGTCTCTGAGCGAGAAGGACATCTTCGT<br>GATTAACCTGAGGGGCAGCTTCAACGACGACCAGAAGGACGCCCTGTACTACGAT<br>GAGGCCAACAGAATTTGGCGGAAGCTGGAAAACATCATGCACAACATCAAAGAGT<br>TCCGGGGCAACAAGACCCGCGAGTACAAGAAGAAAGACGCCCCTAGACTGCCCAG<br>AATCCTGCCTGCTGGCAGAGATGTGTCCGCCTTCTCTAAGCTGATGTACGCCCTGA<br>CCATGTTCCTGGACGGCAAAGAGATCAACGACCTGCTGACCACACTGATCAACAAG<br>TTCGATAACATCCAGAGCTTTCTGAAAGTGATGCCCCTGATCGGCGTGAACGCTAA<br>GTTCGTGGAAGAGTACGCCTTCTTCAAGGACAGCGCCAAGATCGCCGACGAGCTG<br>AGACTGATCAAGAGCTTCGCCAGAATGGGCGAGCCTATCGCCGATGCTAGAAGGG<br>CCATGTACATCGACGCCATTAGAATCCTGGGCACCAACCTGAGCTACGACGAACTG<br>AAGGCTCTGGCTGACACCTTCAGCCTGGACGAGAACGGCAACAAGCTGAAGAAAG<br>GCAAGCACGGCATGCGGAACTTTATCATCAACAACGTGATCAGCAACAAGCGGTTC<br>CACTACCTGATCAGATACGGCGACCCCGCTCATCTGCACGAGATCGCTAAGAACGA<br>GGCCGTGGTCAAGTTCGTGCTGGGCAGAATCGCCGACATCCAGAAGAAGCAGGG<br>ACAGAACGGAAAGAACCAGATCGACCGCTACTACGAGACATGCATCGGCAAGGAC<br>AAGGGCAAGAGCGTGTCCGAGAAAGTGGACGCTCTGACCAAGATCATCACCGGCA<br>TGAACTACGACCAGTTTGACAAAAAGCGGAGCGTGATCGAGGACACCGGCAGAG<br>AGAATGCCGAGAGAGAGAAGTTCAAGAAGATCATCTCCCTGTACCTGACCGTGAT<br>CTACCACATCCTCAAGAACATCGTGAACATCAACGCTCGCTACGTGATCGGCTTCCA<br>CTGCGTGGAAAGAGATGCCCAGCTGTACAAAGAGAAGGGATACGACATCAACCTG<br>AAGAAGCTCGAAGAAAAGGGCTTCAGCAGCGTGACCAAGCTGTGCGCTGGAATCG<br>ACGAGACAGCCCCTGACAAGCGGAAGGACGTGGAAAAAGAGATGGCTGAGAGAG<br>CCAAAGAGAGCATCGACAGCCTGGAAAGCGCCAATCCTAAGCTGTACGCCAATTA<br>CATCAAGTACTCCGACGAGAAGAAAGCCGAAGAGTTCACCAGGCAGATCAACAGA<br>GAGAAGGCCAAGACAGCCCTGAACGCCTACCTGAGAAACACCAAGTGGAATGTGA<br>TCATCCGGGAAGATCTGCTGCGGATCGATAACAAGACCTGCACACTGTTCAGAAAC<br>AAGGCCGTGCACCTGGAAGTCGCGAGATACGTGCACGCCTACATCAACGACATTG<br>CCGAAGTGAACAGCTACTTCCAGCTCTACCACTATATCATGCAGCGGATCATCATG<br>AACGAGCGATATGAGAAGTCCAGCGGCAAGGTGTCCGAGTACTTCGATGCCGTGA<br>ACGATGAGAAGAAGTACAACGACCGGCTGCTGAAACTGCTGTGCGTGCCATTCGG<br>CTACTGCATCCCACGGTTCAAGAACCTGAGCATCGAGGCCCTGTTCGACAGAAACG<br>AGGCTGCCAAATTCGACAAAGAAAAGAAAAAAGTGTCCGGGAACAGCGGATCCAC<br>GCCGCCCAAGAAGAAGAGAAAGGTTGAGGACGGCGAGGGCTTGCAGCTGCCTCC<br>ACTGGAAAGACTGACACTCTCCGGAGCTGCTCCTGCCGCCAAGAAAAAGAAACTC<br>GACTACAAGGACGATGACGACAAGGGATCCGGCGCCACAAACTTCAGCCTGCTTA |

| | |
|---|---|
| | AACAAGCCGGCGACGTCGAGGAAAATCCCGGGCCCGTGTCCAAGGGCGAAGAAC<br>TGATCAAAGAAAACATGCGGATGAAGGTGGTCATGGAAGGCAGCGTGAACGGCC<br>ACCAGTTCAAGTGTACAGGCGAAGGCGAGGGCAGACCTTATGAGGGCGTGCAGA<br>CCATGAGAATCAAAGTGATCGAAGGCGGCCCTCTGCCTTTCGCTTTCGACATCCTG<br>GCCACCAGCTTTATGTACGGCAGCAGAACCTTCATCAAGTACCCCGCTGACATCCC<br>CGATTTCTTCAAGCAGAGCTTCCCCGAGGGCTTCACCTGGGAGAGAGTGACCAGAT<br>ACGAGGATGGCGGCGTGGTCACAGTGACCCAGGACACATCTCTGGAAGATGGCG<br>AGCTGGTGTACAACGTGAAAGTGCGGGGCGTGAACTTCCCCTCTAACGGCCCTGT<br>GATGCAGAAAAAGACCAAAGGCTGGGAGCCCAACACCGAGATGATGTACCCTGCT<br>GATGGCGGCCTGAGAGGCTACACAGATATCGCCCTGAAAGTGGACGGCGGAGGC<br>CATCTGCACTGCAACTTCGTGACCACCTACAGGTCCAAGAAAACCGTGGGCAACAT<br>CAAGATGCCTGGGGTGCACGCCGTGGACCACAGACTGGAAAGAATCGAGGAAAG<br>CGACAACGAGACATACGTGGTGCAGCGCGAAGTGGCCGTGGCCAAGTACTCTAAT<br>CTCGGAGGCGGCATGGACGAGCTGTACAAATGAACGCGTAAATGATTGCAGATCC<br>ACTAGTTCTAGAGCTGCTCGCTTTCTTGCTGTCCAATTTCTATTAAAGGTTCCTTTGT<br>TCCCTAAGTCCAACTACTAAACTGGGGGATATTATGAAGGGCCTTGAGCATCTGGA<br>TTCTGCCTAATAAAAAACATTTATTTTCATTGCGGTACCGCTCGCTTTCTTGCTGTCC<br>AATTTCTATTAAAGGTTCCTTTGTTCCCTAAGTCCAACTACTAAACTGGGGGATATT<br>ATGAAGGGCCTTGAGCATCTGGATTCTGCCTAATAAAAAACATTTATTTTCATTGCG<br>AGCTCGCTGATGGCGCGTCTTAAATAAAAAAAAAAAAAAAAAAAAAAAAAAAAAA<br>AAAAAAAAAAA… |
| SARS-CoV-2-<br>nanoluciferase<br>reporter | ATGGTCTTTACACTGGAAGATTTCGTCGGCGACTGGCGGCAGACAGCTGGCTACA<br>ATCTGGACCAGGTGCTGGAACAAGGCGGCGTGTCCTCTCTGTTTCAGAACCTGGG<br>AGTGTCTGTGACCCCTATCCAGAGAATCGTGCTGAGCGGCGAGAACGGCCTGAAG<br>ATCGACATCCACGTGATCATCCCTTACGAGGGCCTGTCCGGCGATCAGATGGGACA<br>GATCGAGAAGATCTTTAAGGTGGTGTACCCCGTGGACGACCACCACTTCAAAGTG<br>ATCCTGCACTACGGCACCCTGGTCATCGATGGCGTGACCCCAAACATGATCGACTA<br>CTTCGGCAGACCCTACGAGGGAATCGCCGTGTTCGACGGCAAGAAAATCACCGTG<br>ACCGGCACACTGTGGAACGGCAACAAGATCATCGACGAGAGACTGATCAACCCCG<br>ACGGCAGCCTGCTGTTCAGAGTGACAATCAACGGCGTGACAGGCTGGCGGCTGTG<br>CGAAAGAATCCTTGCTGGCAGCGGAAACAGCCACGGCTTCCCACCCGAGGTGGAA<br>GAACAGGCTGCCGGAACCCTGCCTATGAGCTGCGCTCAGGAATCCGGCATGGACA<br>GGCACCCTGCCGCTTGTGCCTCCGCCAGAATCAACGTGTAG |
| Minihelix | GGGCACTCTTCCGTGGTCTGGTGGATAAATTCGCAAGGGTATCATGGCGGACGAC<br>CGGGGTTCGAACCCCGGATCCGGCCGTCCGCCGTGATCCATGCGGTTACCGCCCGC<br>GTGTCGAACCCAGGTGTGCGACGTCAGACAACGGGGGAGCGCTCCTAG |
